# Current best practices in single‐cell RNA‐seq analysis: a tutorial

**DOI:** 10.15252/msb.20188746

**Published:** 2019-06-19

**Authors:** Malte D Luecken, Fabian J Theis

**Affiliations:** ^1^ Institute of Computational Biology Helmholtz Zentrum München German Research Center for Environmental Health Neuherberg Germany; ^2^ Department of Mathematics Technische Universität München Garching bei München Germany

**Keywords:** analysis pipeline development, computational biology, data analysis tutorial, single‐cell RNA‐seq

## Abstract

Single‐cell RNA‐seq has enabled gene expression to be studied at an unprecedented resolution. The promise of this technology is attracting a growing user base for single‐cell analysis methods. As more analysis tools are becoming available, it is becoming increasingly difficult to navigate this landscape and produce an up‐to‐date workflow to analyse one's data. Here, we detail the steps of a typical single‐cell RNA‐seq analysis, including pre‐processing (quality control, normalization, data correction, feature selection, and dimensionality reduction) and cell‐ and gene‐level downstream analysis. We formulate current best‐practice recommendations for these steps based on independent comparison studies. We have integrated these best‐practice recommendations into a workflow, which we apply to a public dataset to further illustrate how these steps work in practice. Our documented case study can be found at https://www.github.com/theislab/single-cell-tutorial. This review will serve as a workflow tutorial for new entrants into the field, and help established users update their analysis pipelines.

## Introduction

In recent years, single‐cell RNA sequencing (scRNA‐seq) has significantly advanced our knowledge of biological systems. We have been able to both study the cellular heterogeneity of zebrafish, frogs and planaria (Briggs *et al*, 2018; Plass *et al*, 2018; Wagner *et al*, 2018) and discover previously obscured cellular populations (Montoro *et al*, 2018; Plasschaert *et al*, 2018). The great potential of this technology has motivated computational biologists to develop a range of analysis tools (Rostom *et al*, 2017). Despite considerable effort being undertaken by the field to ensure the usability of individual tools, a barrier of entry for novices in single‐cell data analysis is the lack of standardization due to the relative immaturity of the field. In this paper, we present a tutorial for scRNA‐seq analysis and outline current best practices to lay a foundation for future analysis standardization.

The challenges to standardization include the growing number of analysis methods (385 tools as of 7 March 2019) and exploding dataset sizes (Angerer *et al*, 2017; Zappia *et al*, 2018). We are continuously finding new ways to use the data at our disposal. For example, it has recently become possible to predict cell fates in differentiation (La Manno *et al*, 2018). While the continuous improvement of analysis tools is beneficial for generating new scientific insight, it complicates standardization.

Further challenges for standardization lie in technical aspects. Analysis tools for scRNA‐seq data are written in a variety of programming languages—most prominently R and Python (Zappia *et al*, 2018). Although cross‐environment support is growing (preprint: Scholz *et al*, 2018), the choice of programming language is often also a choice between analysis tools. Popular platforms such as Seurat (Butler *et al*, 2018), Scater (McCarthy *et al*, 2017), or Scanpy (Wolf *et al*, 2018) provide integrated environments to develop pipelines and contain large analysis toolboxes. However, out of necessity these platforms limit themselves to tools developed in their respective programming languages. By extension, language restrictions also hold true for currently available scRNA‐seq analysis tutorials, many of which revolve around the above platforms (R and bioconductor tools: https://github.com/drisso/bioc2016singlecell and https://hemberg-lab.github.io/scRNA.seq.course/; Lun *et al*, 2016b; Seurat: https://satijalab.org/seurat/get_started.html; Scanpy: https://scanpy.readthedocs.io/en/stable/tutorials.html).

**Figure 1:**
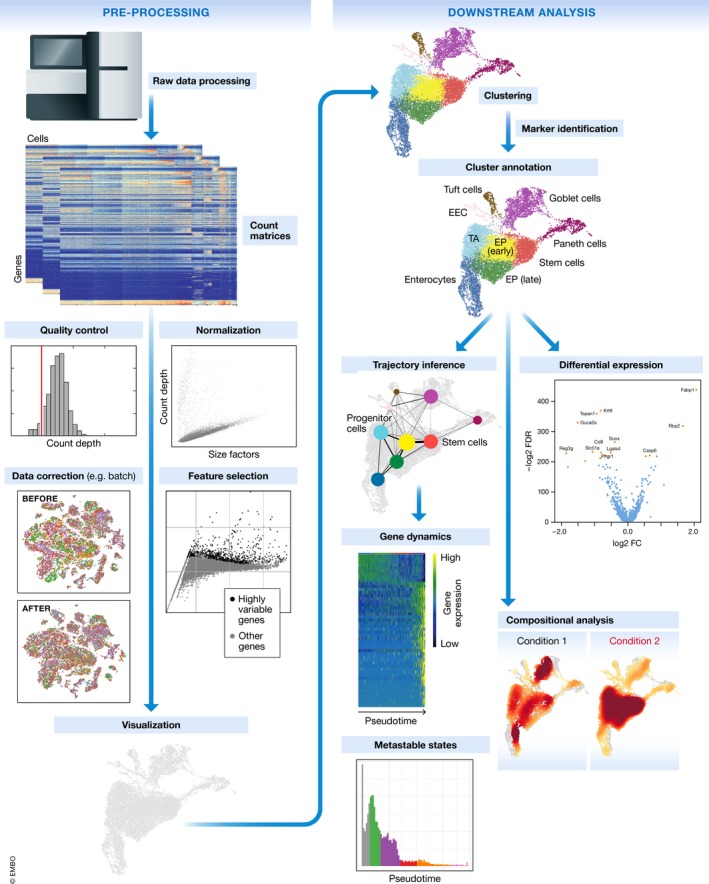
Schematic of a typical single‐cell RNA‐seq analysis workflow Raw sequencing data are processed and aligned to give count matrices, which represent the start of the workflow. The count data undergo pre‐processing and downstream analysis. Subplots are generated using the best‐practices workflow on intestinal epithelium data from Haber *et al* (2017).

Considering the above‐mentioned challenges, instead of targeting a standardized analysis pipeline, we outline current best practices and common tools independent of programming language. We guide the reader through the various steps of a scRNA‐seq analysis pipeline (Fig 1), present current best practices, and discuss analysis pitfalls and open questions. Where best practices cannot be determined due to novelty of the tools and lack of comparisons, we list popular available tools. The outlined steps start from read or count matrices and lead to potential analysis endpoints. Earlier pre‐processing steps are covered in Lun et al (2016b). A detailed case study that integrates the established current best practices is available on our github at: https://github.com/theislab/single-cell-tutorial/. Here, we have applied the current best practices in a practical example workflow to analyse a public dataset. The analysis workflow integrates R and Python tools in a Jupyter–Ipython notebook with rpy2. With the available documentation, it is readily adaptable as a workflow template.

## Pre‐processing and visualization

Raw data generated by sequencing machines are processed to obtain matrices of molecular counts (count matrices) or, alternatively, read counts (read matrices), depending on whether unique molecular identifiers (UMIs) were incorporated in the single‐cell library construction protocol (see Box 1 for an overview of the experimental steps that precede the analysis). Raw data processing pipelines such as Cell Ranger (Zheng *et al*, 2017), indrops (Klein *et al*, 2015), SEQC (Azizi *et al*, 2018), or zUMIs (Parekh *et al*, 2018) take care of read quality control (QC), assigning reads to their cellular barcodes and mRNA molecules of origin (also called “demultiplexing”), genome alignment, and quantification. The resulting read or count matrices have the dimension number of barcodes x number of transcripts. The term “barcode” is used here instead of “cell” as all reads assigned to the same barcode may not correspond to reads from the same cell. A barcode may mistakenly tag multiple cells (doublet) or may not tag any cells (empty droplet/well).

**Box 1: Key elements of an experimental scRNA‐seq workflow**

Generating single‐cell data from a biological sample requires multiple steps. Typical workflows incorporate single‐cell dissociation, single‐cell isolation, library construction, and sequencing. We give a brief overview of these stages here. A more detailed explanation and comparison of different protocols can be found in Ziegenhain *et al* (2017); Macosko *et al* (2015); Svensson *et al* (2017).

 Input material for a single‐cell experiment is typically obtained in the form of biological tissue samples. As a first step, a single‐cell suspension is generated in a process called *single‐cell dissociation* in which the tissue is digested.

 To profile the mRNA in each cell separately, cells must be isolated. *Single‐cell isolation* is performed differently depending on the experimental protocol. While plate‐based techniques isolate cells into wells on a plate, droplet‐based methods rely on capturing each cell in its own microfluidic droplet. In both cases, errors can occur that lead to multiple cells being captured together (*doublets* or *multiplets*), non‐viable cells being captured, or no cell being captured at all (*empty droplets/wells*). Empty droplets are especially common as droplet‐based methods rely on a low concentration flow of input cells to control doublet rates.

 Each well or droplet contains the necessary chemicals to break down the cell membranes and perform *library construction*. Library construction is the process in which the intracellular mRNA is captured, reverse‐transcribed to cDNA molecules and amplified. As cells undergo this process in isolation, the mRNA from each cell can be labelled with a well‐ or droplet‐specific cellular barcode. Furthermore, many experimental protocols also label captured molecules with a *unique molecular identifier (UMI)*. Cellular cDNA is amplified before sequencing to increase its probability of being measured. UMIs allow us to distinguish between amplified copies of the same mRNA molecule and reads from separate mRNA molecules transcribed from the same gene.

 After library construction, cellular cDNA libraries are labelled with cellular barcodes and, depending on the protocol, UMIs. These libraries are pooled together (*multiplexed*) for *sequencing*. Sequencing produces read data, which undergo quality control, grouping based on their assigned barcodes (*demultiplexing*) and alignment in read processing pipelines. For UMI‐based protocols, read data can be further demultiplexed to produce counts of captured mRNA molecules (*count data*).

While read and count data differ in the level of measurement noise, the processing steps in a typical analysis pipeline are the same. For simplicity, we will refer to the data as count matrices in this tutorial. Where results for read and count matrices differ, read matrices are specifically mentioned.

### Quality control

**Figure 2:**
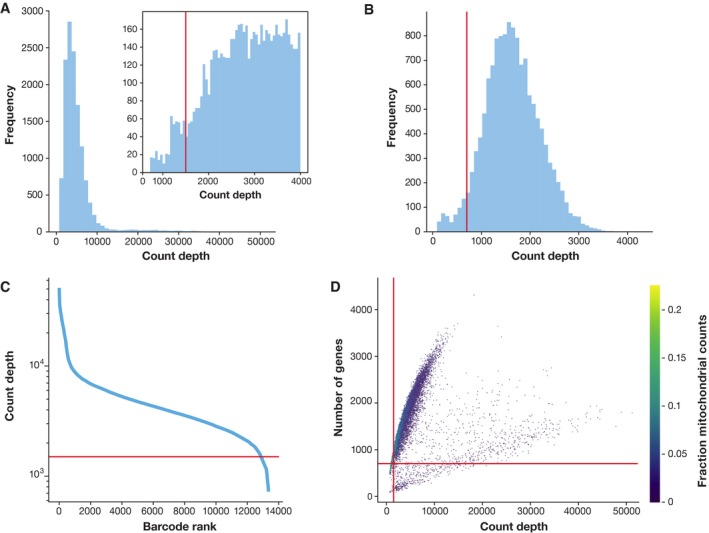
Plots of quality control metrics with filtering decisions for a mouse intestinal epithelium dataset from Haber *et al* (2017) (A) Histograms of count depth per cell. The smaller histogram is zoomed‐in on count depths below 4,000. A threshold is applied here at 1,500 based on the peak detected at around 1,200 counts. (B) Histogram of the number of genes detected per cell. A small noise peak is visible at approx. 400 genes. These cells are filtered out using the depicted threshold (red line) at 700 genes. (C) Count depth distribution from high to low count depths. This visualization is related to the log–log plot shown in Cell Ranger outputs that is used to filter out empty droplets. It shows an “elbow” where count depths start to decrease rapidly around 1,500 counts. (D) Number of genes versus the count depth coloured by the fraction of mitochondrial reads. Mitochondrial read fractions are only high in particularly low count cells with few detected genes. These cells are filtered out by our count and gene number thresholds. Jointly visualizing the count and gene thresholds shows the joint filtering effect, indicating that a lower gene threshold may have sufficed.

Before analysing the single‐cell gene expression data, we must ensure that all cellular barcode data correspond to viable cells. Cell QC is commonly performed based on three QC covariates: the number of counts per barcode (count depth), the number of genes per barcode, and the fraction of counts from mitochondrial genes per barcode (Ilicic *et al*, 2016; Griffiths *et al*, 2018). The distributions of these QC covariates are examined for outlier peaks that are filtered out by thresholding (Fig 2). These outlier barcodes can correspond to dying cells, cells whose membranes are broken, or doublets. For example, barcodes with a low count depth, few detected genes, and a high fraction of mitochondrial counts are indicative of cells whose cytoplasmic mRNA has leaked out through a broken membrane, and thus, only mRNA located in the mitochondria is still conserved (Fig 2). In contrast, cells with unexpectedly high counts and a large number of detected genes may represent doublets. Thus, high‐count depth thresholds are commonly used to filter out potential doublets. Three recent doublet detection tools offer more elegant and potentially better solutions (DoubletDecon: preprint: DePasquale *et al*, 2018; Scrublet: Wolock *et al*, 2019; Doublet Finder: McGinnis *et al*, 2018).

Considering any of these three QC covariates in isolation can lead to misinterpretation of cellular signals. For example, cells with a comparatively high fraction of mitochondrial counts may be involved in respiratory processes. Likewise, other QC covariates also have biological interpretations. Cells with low counts and/or genes may correspond to quiescent cell populations, and cells with high counts may be larger in size. Indeed, molecular counts can differ strongly between cells (see case study on project github). Thus, QC covariates should be considered jointly when univariate thresholding decisions are made (Fig 2D), and these thresholds should be set as permissive as possible to avoid filtering out viable cell populations unintentionally. In future, filtering models that account for multivariate QC dependencies may provide more sensitive QC options.

Datasets that contain heterogeneous mixtures of cell types may exhibit multiple QC covariate peaks. For example, Fig 2D shows two populations of cells with different QC distributions. If no previous filtering step was performed (note that Cell Ranger also performs cell QC), then only the lowest count depth and gene per barcode peak should be considered as non‐viable cells. A further thresholding guideline is the proportion of cells that are filtered out with the chosen threshold. For high‐count filtering, this proportion should not exceed the expected doublet rate.

In addition to checking the integrity of cells, QC steps must also be performed at the level of transcripts. Raw count matrices often include over 20,000 genes. This number can be drastically reduced by filtering out genes that are not expressed in more than a few cells and are thus not informative of the cellular heterogeneity. A guideline to setting this threshold is to use the minimum cell cluster size that is of interest and leaving some leeway for dropout effects. For example, filtering out genes expressed in fewer than 20 cells may make it difficult to detect cell clusters with fewer than 20 cells. For datasets with high dropout rates, this threshold may also complicate the detection of larger clusters. The choice of threshold should scale with the number of cells in the dataset and the intended downstream analysis.

Further QC can be performed on the count data directly. *Ambient gene expression* refers to counts that do not originate from a barcoded cell, but from other lysed cells whose mRNA contaminated the cell suspension prior to library construction. These added ambient counts can distort downstream analysis such as marker gene identification or other differential expression tests especially when levels vary between samples. It is possible to correct for these effects in droplet‐based scRNA‐seq datasets due to the large numbers of empty droplets, which can be used to model ambient RNA expression profiles. The recently developed SoupX (preprint: Young & Behjati, 2018) uses this approach to directly correct the count data. Pragmatic approaches that ignore strongly ambient genes in downstream analysis have also been used to tackle this problem (Angelidis *et al*, 2019).

Quality control is performed to ensure that the data quality is sufficient for downstream analysis. As “sufficient data quality” cannot be determined *a priori*, it is judged based on downstream analysis performance (e.g., cluster annotation). Thus, it may be necessary to revisit quality control decisions multiple times when analysing the data. Often it is beneficial to start with permissive QC thresholds and investigate the effects of these thresholds before going back to perform more stringent QC. This approach is particularly relevant for datasets containing heterogeneous cell populations where cell types or states may be misinterpreted as low‐quality outlier cells. In low‐quality datasets, stringent QC thresholds may be necessary. The quality of a dataset can be determined by experimental QC metrics (see Appendix Supplementary Text S2). In this iterative QC optimization, one should be aware of *data peeking*. QC thresholds should not be adapted to improve the outcome of a statistical test. Instead, QC can be evaluated from the distribution of QC covariates in dataset visualizations and clustering.

**Pitfalls & recommendations:**

- Perform QC by finding outlier peaks in the number of genes, the count depth and the fraction of mitochondrial reads. Consider these covariates jointly instead of separately.
- Be as permissive of QC thresholding as possible, and revisit QC if downstream clustering cannot be interpreted.
- If the distribution of QC covariates differ between samples, QC thresholds should be determined separately for each sample to account for sample quality differences as in Plasschaert *et al* (2018).

### Normalization

Each count in a count matrix represents the successful capture, reverse transcription and sequencing of a molecule of cellular mRNA (Box 1). Count depths for identical cells can differ due to the variability inherent in each of these steps. Thus, when gene expression is compared between cells based on count data, any difference may have arisen solely due to sampling effects. Normalization addresses this issue by e.g. scaling count data to obtain correct relative gene expression abundances between cells.

Many normalization methods exist for bulk gene expression (preprint: Pachter, 2011; Dillies *et al*, 2013). While some of these methods have been applied to scRNA‐seq analysis, sources of variation specific to single‐cell data such as *technical dropouts* (zero counts due to sampling) have prompted the development of scRNA‐seq‐specific normalization methods (Lun *et al*, 2016a; Vallejos *et al*, 2017).

The most commonly used normalization protocol is count depth scaling, also referred to as “counts per million” or CPM normalization. This protocol comes from bulk expression analysis and normalizes count data using a so‐called size factor proportional to the count depth per cell. Variations of this method scale the size factors with different factors of 10, or by the median count depth per cell in the dataset. CPM normalization assumes that all cells in the dataset initially contained an equal number of mRNA molecules and count depth differences arise only due to sampling. This assumption is shared with the downsampling protocol, which is the process of randomly sampling reads or counts from the data to leave all cells with a pre‐specified number of counts or fewer. While downsampling throws away data, it also increases technical dropout rates which CPM and other global scaling normalization methods do not. Thus, downsampling can deliver a more realistic representation of what cellular expression profiles would look like at similar count depths.

As single‐cell datasets typically consist of heterogeneous cell populations with varying sizes and molecule counts, more complex normalization methods are usually appropriate. For example, Weinreb *et al* (2018) used a simple extension of CPM that excludes genes that account for at least 5% of the total counts in any cell, when calculating their size factors. This approach allows for molecular count variability in few highly expressed genes. More cellular heterogeneity is permitted by Scran's pooling‐based size factor estimation method (Lun et al, 2016a). Here, size factors are estimated based on a linear regression over genes, after cells are pooled to avoid technical dropout effects. This method limits variability to fewer than 50% of genes being differentially expressed between cells, and is consistently a top‐performing normalization method in independent comparisons. Scran has been shown to perform better than other tested normalization methods for batch correction (Buttner *et al*, 2019) and differential expression analysis (preprint: Vieth *et al,* 2019). The method was also shown to give robust size factor estimates in a small‐scale comparison from the original authors (Vallejos *et al*, 2017).

CPM, high‐count filtering CPM, and scran use linear, global scaling to normalize count data. Non‐linear normalization methods, which can account for more complex unwanted variation, also exist (Cole *et al*, 2019). Many such methods involve the parametric modelling of count data. For example, Mayer *et al* (2018) fit a negative binomial model to count data, using technical covariates such as the read depth and the number of counts per gene to fit the model parameters. The residuals of the model fit serve as a normalized quantification of gene expression. Such an approach can combine technical and biological data correction (e.g. batch correction or correction for cell cycle effects) with count depth normalization. Non‐linear normalization methods have been shown to outperform global scaling methods especially in situations with strong batch effects (Cole *et al*, 2019). Thus, non‐linear normalization methods are particularly relevant for plate‐based scRNA‐seq data, which tend to have batch effects between plates. Furthermore, plate‐based data can exhibit larger variations in count depths per cell than droplet‐based data (Svensson *et al*, 2017). While non‐linear normalization methods, or alternative approaches such as downsampling, appear better suited to these conditions, comparative studies are needed to confirm this hypothesis. In this tutorial, we prefer to separate the normalization and data correction (batch correction, noise correction, etc.) steps to emphasize different processing stages of the data (see “Stages of pre‐processed data” section). Thus, we focus on global scaling normalization methods.

We cannot expect that a single normalization method is appropriate for all types of scRNA‐seq data. For example, Vieth *et al* (2017) showed that read and count data are best fit by different models. Indeed Cole *et al* (2019) find that different normalization methods perform optimally for different datasets and argue that their *scone* tool should be used to select the appropriate normalization method for a specific dataset. Furthermore, scRNA‐seq techniques can be divided into full‐length and 3′ enrichment methods (Svensson *et al*, 2017; Ziegenhain *et al*, 2017). Data from full‐length protocols may benefit from normalization methods that take into account gene length (e.g. Patel *et al*, 2014; Kowalczyk *et al*, 2015; Soneson & Robinson, 2018), while 3′ enrichment data do not. A commonly used normalization method for full‐length scRNA‐seq data is TPM normalization (Li *et al*, 2009), which comes from bulk RNA‐seq analysis.

In the same way that cellular count data can be normalized to make them comparable between cells, gene counts can be scaled to improve comparisons between genes. Gene normalization constitutes scaling gene counts to have zero mean and unit variance (z scores). This scaling has the effect that all genes are weighted equally for downstream analysis. There is currently no consensus on whether or not to perform normalization over genes. While the popular Seurat tutorials (Butler *et al*, 2018) generally apply gene scaling, the authors of the Slingshot method opt against scaling over genes in their tutorial (Street *et al*, 2018). The preference between the two choices revolves around whether all genes should be weighted equally for downstream analysis, or whether the magnitude of expression of a gene is an informative proxy for the importance of the gene. In order to retain as much biological information as possible from the data, we opt to refrain from scaling over genes in this tutorial.

After normalization, data matrices are typically log(x+1)‐transformed. This transformation has three important effects. Firstly, distances between log‐transformed expression values represent log fold changes, which are the canonical way to measure changes in expression. Secondly, log transformation mitigates (but does not remove) the mean–variance relationship in single‐cell data (Brennecke *et al*, 2013). Finally, log transformation reduces the skewness of the data to approximate the assumption of many downstream analysis tools that the data are normally distributed. While scRNA‐seq data are not in fact log‐normally distributed (Vieth *et al*, 2017), these three effects make the log transformation a crude, but useful tool. This usefulness is highlighted by downstream applications for differential expression testing (Finak *et al*, 2015; Ritchie *et al*, 2015) or batch correction (Johnson *et al*, 2006; Buttner *et al*, 2019) that use log transformation for these purposes. It should however be noted that log transformation of normalized data can introduce spurious differential expression effects into the data (preprint: Lun, 2018). This effect is particularly pronounced when normalization size factor distributions differ strongly between tested groups.

**Pitfalls & recommendations:**

- We recommend scran for normalization of non‐full‐length datasets. An alternative is to evaluate normalization approaches via *scone* especially for plate‐based datasets. Full‐length scRNA‐seq protocols can be corrected for gene length using bulk methods.
- There is no consensus on scaling genes to 0 mean and unit variance. We prefer not to scale gene expression.
- Normalized data should be log(x+1)‐transformed for use with downstream analysis methods that assume data are normally distributed.

### Data correction and integration

Normalization as described above attempts to remove the effects of count sampling. However, normalized data may still contain unwanted variability. Data correction targets further technical and biological covariates such as batch, dropout, or cell cycle effects. These covariates are not always corrected for. Instead, the decision of which covariates to consider will depend on the intended downstream analysis. We propose to consider correction for biological and technical covariates separately as these are used for different purposes and present unique challenges.

#### Regressing out biological effects

While correcting for technical covariates may be crucial to uncovering the underlying biological signal, correction for biological covariates serves to single out particular biological signals of interest. The most common biological data correction is to remove the effects of the cell cycle on the transcriptome. This data correction can be performed by a simple linear regression against a cell cycle score as implemented in the Scanpy and Seurat platforms (Butler *et al*, 2018; Wolf *et al*, 2018) or in specialized packages with more complex mixture models such as scLVM (Buettner *et al*, 2015) or f‐scLVM (Buettner *et al*, 2017). Lists of marker genes to compute cell cycle scores are obtained from the literature (Macosko *et al*, 2015). These methods can also be used to regress out other known biological effects such as mitochondrial gene expression, which is interpreted as an indication of cell stress.

Several aspects should be considered prior to correcting data for biological effects. Firstly, correcting for biological covariates is not always helpful to interpret scRNA‐seq data. While removing cell cycle effects can improve the inference of developmental trajectories (Buettner *et al*, 2015; Vento‐Tormo *et al*, 2018), cell cycle signals can also be informative of the biology. For example, proliferating cell populations can be identified based on cell cycle scores (see case study on project github). Also, biological signals must be understood in context. Given that biological processes occur within the same organism, there exist dependencies between these processes. Thus, correcting for one process may unintentionally mask the signal of another. Finally, it has been argued that variation in cell size accounts for the transcriptomic effect generally attributed to the cell cycle (McDavid *et al*, 2016). Thus, correcting for cell size via normalization, or dedicated tools such as cgCorrect (Blasi *et al*, 2017), also partially corrects for cell cycle effects in scRNA‐seq data.

#### Regressing out technical effects

The variants of regression models used to regress out biological covariates can also be applied to technical covariates. The most prominent technical covariates in single‐cell data are count depth and batch. Although normalization scales count data to render gene counts comparable between cells, a count depth effect often remains in the data. This count depth effect can be both a biological and a technical artefact. For example, cells may differ in size and therefore in mRNA molecule counts. Yet, technical count effects may remain after normalization as no scaling method can infer the expression values of genes that were not detected due to poor sampling. Regressing out count depth effects can improve the performance of trajectory inference algorithms, which rely on finding transitions between cells (see case study on project github). When correcting for multiple covariates (e.g. cell cycle and count depth), the regression should be performed over all covariates in a single step to account for dependence between covariates.

An alternative to regression‐based strategies for removing count effects is to use a more rigorous normalization procedure such as downsampling or non‐linear normalization methods (see “Normalization” section). These approaches may be particularly relevant for plate‐based scRNA‐seq datasets where the larger variation of count depths per cell can mask the heterogeneity between cells.

#### Batch effects and data integration

Batch effects can occur when cells are handled in distinct groups. These groups can consist of cells on different chips, cells in different sequencing lanes or cells harvested at different time points. The differing environments experienced by the cells can have an effect on the measurement of the transcriptome or on the transcriptome itself. The resulting effects exist on multiple levels: between groups of cells in an experiment, between experiments performed in the same laboratory or between datasets from different laboratories. Here, we distinguish between the first and the last two scenarios. Correcting for batch effects between samples or cells in the same experiment is the classical scenario known as *batch correction* from bulk RNA‐seq. We distinguish this from the integration of data from multiple experiments, which we call *data integration*. While batch effects are typically corrected using linear methods, non‐linear approaches are used for data integration.

**Figure 3:**
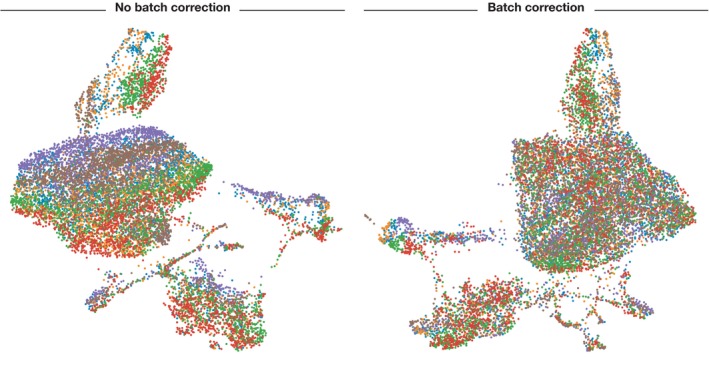
UMAP visualization before and after batch correction Cells are coloured by sample of origin. Separation of batches is clearly visible before batch correction and less visible afterwards. Batch correction was performed using ComBat on mouse intestinal epithelium data from Haber *et al* (2017).

A recent comparison of classical batch correction methods has revealed that ComBat (Johnson *et al*, 2006) performs well also for single‐cell experiments of low‐to‐medium complexity (Buttner *et al*, 2019). ComBat consists of a linear model of gene expression where the batch contribution is taken into account both in the mean and the variance of the data (Fig 3). Irrespective of computational methods, the best method of batch correction is pre‐empting the effect and avoiding it altogether by clever experimental design (Hicks *et al*, 2017). Batch effects can be avoided by pooling cells across experimental conditions and samples. Using strategies such as cell tagging (preprint: Gehring *et al*, 2018), or via genetic variation (Kang *et al*, 2018), it is possible to demultiplex cells that were pooled in the experiment.

In comparison with batch correction, the additional challenge that data integration methods face revolves around compositional differences between datasets. When estimating batch effects, ComBat uses all cells in a batch to fit batch parameters. This approach will confound the batch effect with biological differences between cell types or states that are not shared among datasets. Data integration methods such as Canonical Correlation Analysis (CCA; Butler *et al*, 2018), Mutual Nearest Neighbours (MNN; Haghverdi *et al*, 2018), Scanorama (preprint: Hie *et al*, 2018), RISC (preprint: Liu *et al*, 2018), scGen (preprint: Lotfollahi *et al*, 2018), LIGER (preprint: Welch *et al*, 2018), BBKNN (preprint: Park *et al*, 2018), and Harmony (preprint: Korsunsky *et al*, 2018) have been developed to overcome this issue. While data integration methods can also be applied to simple batch correction problems, we recommend to be wary of over‐correction given the increased degrees of freedom of non‐linear data integration approaches. For example, MNN was shown to be outperformed by ComBat in the simpler batch correction setting (Buttner *et al*, 2019). Further comparison studies between data integration and batch correction methods are needed to assess how generally these methods can be applied.

#### Expression recovery

A further type of technical data correction is *expression recovery* (also *denoising* or *imputation*). Measurements of single‐cell transcriptomes contain various sources of noise (Grün *et al*, 2014; Kharchenko *et al*, 2014; Hicks *et al*, 2017). A particularly prominent aspect of this noise is dropout. Inferring dropout events, replacing these zeros with appropriate expression values, and reducing the noise in the dataset have been the target of several recent tools (MAGIC: van Dijk *et al*, 2018; DCA: Eraslan *et al*, 2019; scVI: Lopez *et al*, 2018; SAVER: Huang *et al*, 2018; scImpute: Li & Li, 2018). Performing expression recovery has been shown to improve the estimation of gene–gene correlations (van Dijk *et al*, 2018; Eraslan *et al*, 2019). Furthermore, this step can be integrated with normalization, batch correction and other downstream analysis as implemented in the scVI tool (Lopez *et al*, 2018). While most data correction methods take normalized data as input, some expression recovery methods are based on expected negative binomial noise distributions and therefore run on raw count data. When applying expression recovery, one should take into consideration that no method is perfect. Thus, any method may over‐ or under‐correct noise in the data. Indeed, false correlation signals have been reported as a result of expression recovery (Andrews & Hemberg, 2018). Given the difficulty of assessing successful expression recovery in a practical application, this scenario represents a challenge to the user pondering whether or not to denoise their data. Furthermore, scalability to large datasets is still an issue for currently available expression recovery methods. There is currently no consensus on how denoised data should be used in the light of these considerations (see “Stages of processed data” section). A prudent approach would be to use expression recovery only for visual display of data rather than to generate hypotheses during exploratory data analysis. Thorough experimental validation is particularly important here.

**Pitfalls & recommendations:**

- Regress out biological covariates only for trajectory inference and if other biological processes of interest are not masked by the regressed out biological covariate.
- Regress out technical and biological covariates jointly rather than serially.
- Plate‐based dataset pre‐processing may require regressing out counts, normalization via non‐linear normalization methods or downsampling.
- We recommend performing batch correction via ComBat when cell type and state compositions between batches are consistent
- Data integration and batch correction should be performed by different methods. Data integration tools may over‐correct simple batch effects.
- Users should be cautious of signals found only after expression recovery. Exploratory analysis may be best performed without this step.

### Feature selection, dimensionality reduction and visualization

A human single‐cell RNA‐seq dataset can contain expression values for up to 25,000 genes. Many of these genes will not be informative for a given scRNA‐seq dataset, and many genes will mostly contain zero counts. Even after filtering out these zero count genes in the QC step, the feature space for a single‐cell dataset can have over 15,000 dimensions. To ease the computational burden on downstream analysis tools, reduce the noise in the data, and to visualize the data, one can use several approaches to reduce the dimensionality of the dataset.

#### Feature selection

**Figure EV1:**
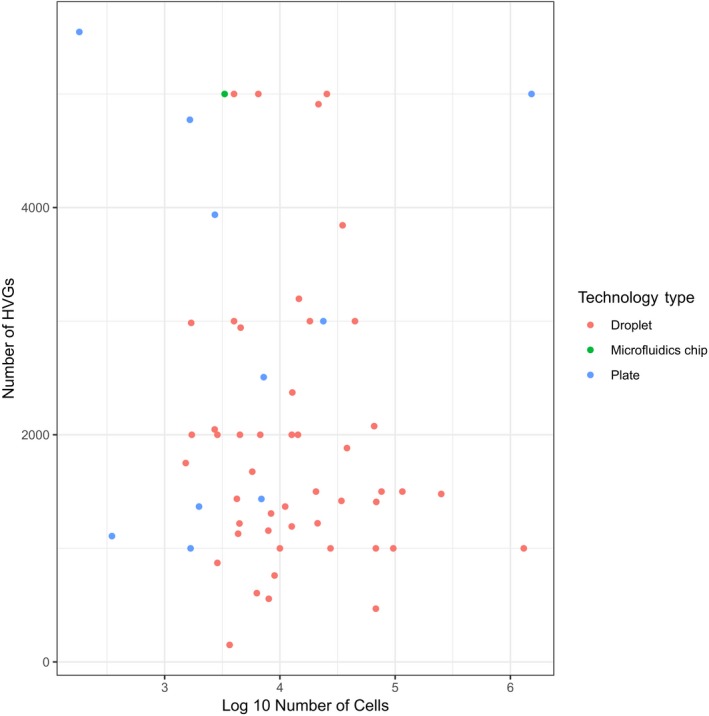
The number of highly variable genes (HVGs) used for datasets of different sizes The data were obtained by a brief manual survey of recent scRNA‐seq analysis papers. The plotted data, along with further information on scRNA‐seq technology, publication year, reference and the number of reads per cell, are available in Dataset EV1.

The first step of reducing the dimensionality of scRNA‐seq datasets commonly is feature selection. In this step, the dataset is filtered to keep only genes that are “informative” of the variability in the data. Thus, highly variable genes (HVGs) are often used (Brennecke *et al*, 2013). Depending on the task and the complexity of the dataset, typically between 1,000 and 5,000 HVGs are selected for downstream analysis (see Fig EV1 and Dataset EV1). Preliminary results from Klein *et al* (2015) suggest that downstream analysis is robust to the exact choice of the number of HVGs. While varying the number of HVGs between 200 and 2,400, the authors reported similar low‐dimensional representations in the PCA space. Based on this result, we prefer to err on the side of higher numbers of HVGs.

A simple, yet popular, method of selecting HVGs is implemented in both Scanpy and Seurat. Here, genes are binned by their mean expression, and the genes with the highest variance‐to‐mean ratio are selected as HVGs in each bin. There exist different flavours of this algorithm that expect either count data (Seurat) or log‐transformed data (Cell Ranger). Optimally, HVGs should be selected after technical data correction to avoid selecting genes that are highly variable only due to, e.g., batch effects. Other methods for HVG selection are reviewed in Yip *et al* (2018).

#### Dimensionality reduction

After feature selection, the dimensions of single‐cell expression matrices can be further reduced by dedicated dimensionality reduction algorithms. These algorithms embed the expression matrix into a low‐dimensional space, which is designed to capture the underlying structure in the data in as few dimensions as possible. This approach works as single‐cell RNA‐seq data are inherently low‐dimensional (Heimberg *et al*, 2016). In other words, the biological manifold on which cellular expression profiles lie can be sufficiently described by far fewer dimensions than the number of genes. Dimensionality reduction aims to find these dimensions.

There are two main objectives of dimensionality reduction methods: *visualization* and *summarization*. Visualization is the attempt to optimally describe the dataset in two or three dimensions. These reduced dimensions are used as coordinates on a scatter plot to obtain a visual representation of the data. Summarization does not prescribe the number of output components. Instead, higher components become less important for describing the variability present in the data. Summarization techniques can be used to reduce the data to its essential components by finding the inherent dimensionality of the data, and are thus helpful for downstream analysis. While a 2‐dimensional visualization output should not be used to summarize a dataset, a summarization method can be used to visualize the data using the leading reduced components. However, a dedicated visualization technique will typically provide a better representation of the variability.

**Figure 4:**
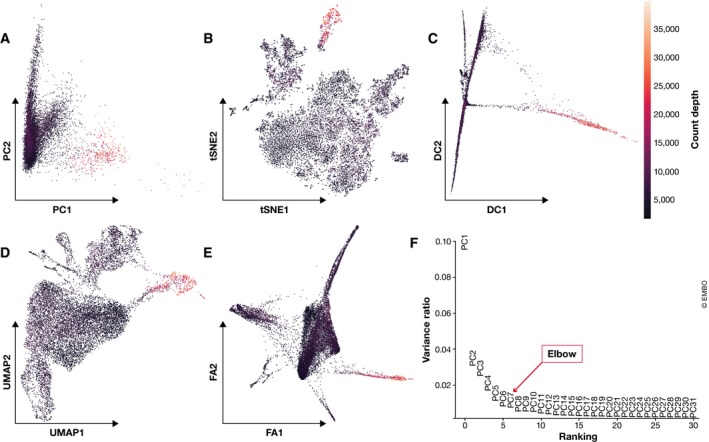
Common visualization methods for scRNA‐seq data Mouse intestinal epithelium regions data from Haber *et al* (2017) visualized on the first two components for: (A) PCA, (B) t‐SNE, (C) diffusion maps, (D) UMAP and (E) A force‐directed graph layout via ForceAtlas2. Cells are coloured by count depth. (F) Variance explained by the first 31 principal components (PCs). The “elbow” of this plot, which is used to select relevant PCs to analyse the dataset, lies between PCs 5 and 7.

Reduced dimensions are generated through linear or non‐linear combinations of feature space dimensions (gene expression vectors). Especially in the non‐linear case, the interpretability of the reduced dimensions is sacrificed in this process. An example application of some commonly used dimensionality reduction methods is shown in Fig 4. With a growing list of methods to choose from, it is out of the scope of this tutorial to review these methods in detail. Rather, we briefly outline the practical considerations that may aid users in choosing between common dimensionality reduction methods. A more detailed review of dimensionality reduction for single‐cell analysis can be found in Moon *et al* (2018).

Two popular dimensionality reduction techniques that are principally summarization methods are principal component analysis (PCA; Pearson, 1901) and diffusion maps (Coifman *et al*, 2005) which were popularized for single‐cell analysis by Haghverdi *et al* (2015). Principal component analysis is a linear approach that generates reduced dimensions by maximizing the captured residual variance in each further dimension. While PCA does not capture the structure of the data in few dimensions as well as non‐linear methods, it is the basis of many currently available analysis tools for clustering or trajectory inference. Indeed, PCA is commonly used as a pre‐processing step for non‐linear dimensionality reduction methods. Typically, PCA summarizes a dataset via its top N principal components, where N can be determined by “elbow” heuristics (see Fig 4F) or the permutation‐test‐based jackstraw method (Chung & Storey, 2015; Macosko *et al*, 2015). The simple linearity of PCA has the advantage that distances in reduced dimensional space have a consistent interpretation in all regions of this space. Thus, we can correlate quantities of interest with principal components to assess their importance. For example, principal components can be projected onto technical nuisance covariates to investigate the performance of QC, data correction and normalization steps (Buttner *et al*, 2019), or show the importance of genes in the dataset (Chung & Storey, 2015). Diffusion maps are a non‐linear data summarization technique. As diffusion components emphasize transitions in the data, they are principally used when continuous processes such as differentiation are of interest. Typically, each diffusion component (i.e. diffusion map dimension) highlights the heterogeneity of a different cell population.

#### Visualization

For visualization purposes, it is standard practice to use non‐linear dimensionality reduction methods (Fig 4). The most common dimensionality reduction method for scRNA‐seq visualization is the t‐distributed stochastic neighbour embedding (t‐SNE; van der Maaten & Hinton, 2008). t‐SNE dimensions focus on capturing local similarity at the expense of global structure. Thus, these visualizations may exaggerate differences between cell populations and overlook potential connections between these populations. A further difficulty is the choice of its perplexity parameter, as t‐SNE graphs may show strongly different numbers of clusters depending on its value (Wattenberg *et al*, 2016). Common alternatives to t‐SNE are the Uniform Approximation and Projection method (UMAP; preprint: McInnes & Healy, 2018) or graph‐based tools such as SPRING (Weinreb *et al*, 2018). UMAP and SPRING's force‐directed layout algorithm ForceAtlas2 arguably represent the best approximation of the underlying topology (Wolf *et al*, 2019, Supplemental Note 4). What sets UMAP apart in this comparison is its speed and ability to scale to large numbers of cells (Becht *et al*, 2018). Thus, in the absence of particular biological questions, we regard UMAP as best practice for exploratory data visualization. Moreover, UMAP can also summarize data in more than two dimensions. While we are not aware of any applications of UMAP for data summarization, it may prove a suitable alternative to PCA.

An alternative to classical visualization on the cell level is partition‐based graph abstraction (PAGA; Wolf *et al*, 2019). This tool has been shown to adequately approximate the topology of the data while coarse‐graining the visualization using clusters. In combination with any of the above visualization methods, PAGA produces coarse‐grained visualizations, which can simplify the interpretation of single‐cell data especially with large numbers of cells.

**Pitfalls & recommendations:**

- We recommend selecting between 1,000 and 5,000 highly variable genes depending on dataset complexity.
- Feature selection methods that use gene expression means and variances cannot be used when gene expression values have been normalized to zero mean and unit variance, or when residuals from model fitting are used as normalized expression values. Thus, one must consider what pre‐processing to perform before selecting HVGs.
- Dimensionality reduction methods should be considered separately for summarization and visualization.
- We recommend UMAP for exploratory visualization; PCA for general purpose summarization; and diffusion maps as an alternative to PCA for trajectory inference summarization.
- PAGA with UMAP is a suitable alternative to visualize particularly complex datasets.

### Stages of pre‐processed data

While we have outlined common pre‐processing steps in scRNA‐seq as a sequential pipeline above, downstream analyses often prefer to take different levels of pre‐processed data, and it is recommended to adapt pre‐processing depending on the downstream application. To clarify this situation to a new user, we delineated pre‐processing into five stages of data processing: (i) raw data, (ii) normalized data, (iii) corrected data, (iv) feature‐selected data, and (v) dimensionality‐reduced data. These stages of data processing are grouped into three pre‐processing layers: measured data, corrected data, and reduced data. Cell and gene QC should always be performed and is therefore omitted from this characterization. While the order of the layers represent the typical workflow in scRNA‐seq analysis, it is also possible to skip layers or have slight alterations in the order of processing stages. For example, data correction may not be necessary for single batch datasets. In Table 1, we summarize the appropriate downstream applications for each layer of pre‐processed data.

**Table 1:** Stages of data processing and appropriate downstream applications

Pre‐processing layer	Stage of data processing	Appropriate applications
Measured	1) Raw	Statistical testing (Differential expression: marker genes, genes over condition, genes over time)
	2) Normalized (+ log transformed)	
Corrected	3.1) Corrected (technical correction)	Visual comparison of data (plotting)
	3.2) Corrected (biological correction)	Pre‐processing for trajectory inference
Reduced	4) Feature selected	Visualization, trajectory inference
	5) Dimensionality reduced (summarized)	Visualization, clustering, KNN graph inference, trajectory inference

The stages of pre‐processing in Table 1 are divided into three groups: measured data, corrected data and reduced data. We define *measured data* as raw data and processed data that retain the structure of zeros. By scaling count data with a cell‐specific factor, global scaling normalization methods retain zero expression values even after log(x+1)‐transformation. In contrast, correcting data for unwanted variability replaces zero expression values. The *corrected data* layer represents the “cleanest” version of the data, which is the closest approximation of the underlying biological signal. We call the final pre‐processing layer *reduced data*. This data layer emphasizes dominant aspects of the data, which can be described using a reduced set of features.

The aforementioned characteristics determine the suitability of the pre‐processed data for particular downstream applications. As the final pre‐processing stage, reduced data would be the natural candidate for a broadly applicable data layer. However, testing for differential expression is only biologically interpretable in gene space, which is not (fully) represented in reduced data. The strength of reduced data lies in the summarization of the biology and the reduction of noise, which can mask biological signals. Thus, reduced data are used for exploratory methods that require data summaries (visualization, neighbourhood graph inference, clustering) and for computationally complex downstream analysis tools (trajectory inference). Indeed, many trajectory inference methods incorporate dimensionality reduction in the tools themselves.

The expression profiles of individual genes can only be compared in gene space, which is captured in measured and corrected data. Comparison of expression profiles can be performed visually and statistically. We argue that visual and statistical comparison should be performed on different data layers. For visual inspection of gene expression, corrected data are most appropriate. Should raw data be presented for visual comparison, the user is required to inherently understand the biases in the data in order to interpret the results. Corrected data facilitate this interpretation. However, one should consider corrected data for technical and biological covariates separately here. While correction for biological covariates may increase the strength of a particular biological signal, it produces a less accurate representation of the underlying biology and will mask other signals that may be relevant. Thus, biologically corrected data are appropriate mainly for analysis tools that focus on particular biological processes such as trajectory inference methods.

**Figure EV2:**
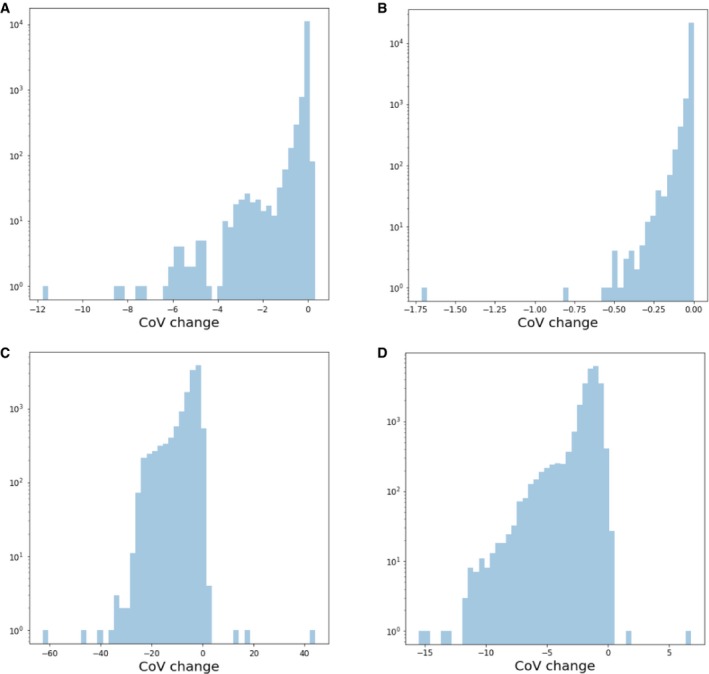
Change in coefficient of variation (CoV) of gene expression data upon batch correction and denoising Negative values represent a reduction in CoV upon data correction. The top row shows CoV changes upon ComBat batch correction for (A) mouse intestinal epithelium (mIE) and (B) mouse embryonic stem cell (mESC) data. The lower row depicts CoV changes upon DCA denoising for (C) mIE and (D) mESC data. mIE data were obtained from Haber *et al* (2017) and mESC from Klein *et al* (2015).

Statistical comparison of gene expression is most appropriate on the measured data layer. No perfect data correction method exists for denoising, batch correction or correcting for other sources of variation. Thus, data correction methods inevitably over‐ or under‐correct the data and therefore alter the variance of at least some gene expression profiles in an unintended way. Statistical tests of gene expression rely on assessing the background variance as a null model for noise in the data. As data correction tends to reduce background variation (Fig EV2), genes whose background variation is over‐corrected by data correction methods will be more likely assessed as significantly differentially expressed. Furthermore, certain data correction methods (e.g. ComBat) interpret expression signals that do not conform to the experimental design as noise, which is subsequently removed from the data. In addition to an underestimation of the noise, this optimization of the experimental design signal can lead to an overestimation of the effect size. In the light of these considerations, using measured data as input, as opposed to corrected data, constitutes a more conservative approach to differential testing. With measured data, technical covariates can and should be taken into consideration in the differential testing model.

The above view is supported by a recent comparison of scRNA‐seq differential expression methods, which uses only raw and normalized data as input (Soneson & Robinson, 2018). The normalized data used in this study revolve only around global scaling methods. However, many currently available non‐linear normalization methods blur the line between normalization and data correction (see “Normalization” section). Such normalized data may no longer be appropriate as input for differential testing.

**Pitfalls & recommendations:**

- Use measured data for statistical testing, corrected data for visual comparison of data and reduced data for other downstream analysis based on finding the underlying biological data manifold.

#### Downstream analysis

After pre‐processing, methods that we call *downstream analysis* are used to extract biological insights and describe the underlying biological system. These descriptions are obtained by fitting interpretable models to the data. Examples of such models are groups of cells with similar gene expression profiles representing cell‐type clusters; small changes in gene expression between similar cells denoting continuous (differentiation) trajectories; or genes with correlated expression profiles indicating co‐regulation.

**Figure 5:**
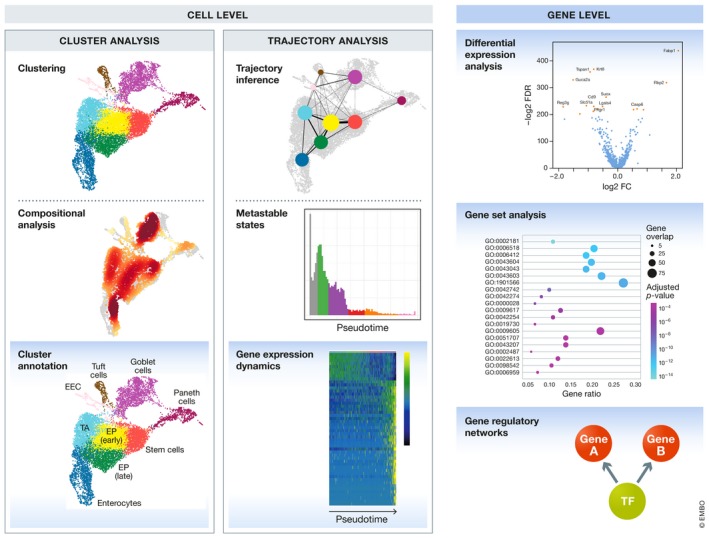
Overview of downstream analysis methods Methods are divided into cell‐ and gene‐level analysis. Cell‐level analysis approaches are again subdivided into cluster and trajectory analysis branches, which include also gene‐level analysis methods. All methods with a blue background are gene‐level approaches.

Downstream analysis can be divided into cell‐ and gene‐level approaches as shown in Fig 5. Cell‐level analysis typically focuses on the description of two structures: clusters and trajectories. These structures can in turn be analysed on the cell and the gene level leading to cluster analysis and trajectory analysis methods. Broadly, cluster analysis methods attempt to explain the heterogeneity in the data based on a categorization of cells into groups. In contrast, in trajectory analysis the data are regarded as a snapshot of a dynamic process. Trajectory analysis methods investigate this underlying process.

Here, we describe cell‐ and gene‐level cluster and trajectory analysis tools before detailing gene‐level analyses that are performed independently of these cellular structures.

### Cluster analysis

#### Clustering

Organizing cells into clusters is typically the first intermediate result of any single‐cell analysis. Clusters allow us to infer the identity of member cells. Clusters are obtained by grouping cells based on the similarity of their gene expression profiles. Expression profile similarity is determined via distance metrics, which often take dimensionality‐reduced representations as input. A common example of similarity scoring is Euclidean distances that are calculated on the PC‐reduced expression space. Two approaches exist to generate cell clusters from these similarity scores: clustering algorithms and community detection methods.

Clustering is a classical unsupervised machine learning problem, based directly on a distance matrix. Cells are assigned to clusters by minimizing intracluster distances or finding dense regions in the reduced expression space. The popular *k*‐means clustering algorithm divides cells into *k* clusters by determining cluster centroids and assigning cells to the nearest cluster centroid. Centroid positions are iteratively optimized (MacQueen, 1967). This approach requires an input of the number of clusters expected, which is usually unknown and must be calibrated heuristically. Applications of k‐means to single‐cell data vary in the distance metrics used. Alternatives to standard Euclidean distances include cosine similarity (Haghverdi *et al*, 2018), correlation‐based distance metrics (Kim *et al*, 2018) or the SIMLR method, which learns a distance metric for each dataset using Gaussian kernels (Wang *et al*, 2017). A recent comparison has suggested that correlation‐based distances may outperform other distance metrics when used with k‐means or as the basis for Gaussian kernels (Kim *et al*, 2018).

Community detection methods are graph‐partitioning algorithms and thus rely on a graph representation of single‐cell data. This graph representation is obtained using a *K*‐Nearest Neighbour approach (KNN graph). Cells are represented as nodes in the graph. Each cell is connected to its *K* most similar cells, which are typically obtained using Euclidean distances on the PC‐reduced expression space. Depending on the size of the dataset, *K* is commonly set to be between 5 and 100 nearest neighbours. The resulting graph captures the underlying topology of the expression data (Wolf *et al*, 2019). Densely sampled regions of expression space are represented as densely connected regions of the graph. These dense regions are detected using community detection methods. Community detection is often faster than clustering as only neighbouring cell pairs have to be considered as belonging to the same cluster. This approach thus greatly reduces the search space for possible clusters.

After the pioneering PhenoGraph method (Levine *et al*, 2015), the standard approach to clustering single‐cell datasets has become multi‐resolution modularity optimization (Newman & Girvan, 2004; Reichardt & Bornholdt, 2006) as implemented in the Louvain algorithm (Blondel *et al*, 2008) on single‐cell KNN graphs. This method is the default clustering method implemented in the Scanpy and Seurat single‐cell analysis platforms. It has been shown to outperform other clustering methods for single‐cell RNA‐seq data (Duò *et al*, 2018; Freytag *et al*, 2018), and flow and mass cytometry data (Weber & Robinson, 2016). Conceptually, the Louvain algorithm detects communities as groups of cells that have more links between them than expected from the number of links the cells have in total. The optimized modularity function includes a resolution parameter, which allows the user to determine the scale of the cluster partition. By subsetting the KNN graph, it is also possible to subcluster only particular clusters. Such subclustering can allow the user to identify cell states within cell‐type clusters (Wagner *et al*, 2016), but may also lead to patterns that arise only from noise in the data.

**Pitfalls & recommendations:**

- We recommend clustering by Louvain community detection on a single‐cell KNN graph.
- Clustering does not have to be performed at a single resolution. Subclustering particular cell clusters is a valid approach to focus on more detailed substructures in a dataset.

#### Cluster annotation

On a gene level, clustered data are analysed by finding the gene signatures of each cluster. These so‐called *marker genes* characterize the cluster and are used to annotate it with a meaningful biological label. This label represents the identity of cells within the cluster. As any clustering algorithm will produce a partition of the data, the validity of the identified clusters can only be determined by successful annotation of the represented biology.

While it may be tempting to assume that the clusters detected in single‐cell data represent cell types, there are several axes of variation that determine cellular identity (Wagner *et al*, 2016; Clevers *et al*, 2017). Firstly, it is not always clear what constitutes a cell type. For example, while “T cells” may be a satisfactory label of a cell type to some, others may look for T‐cell subtypes within a dataset and distinguish between CD4^+^ and CD8^+^ T cells (Wagner *et al*, 2016; Clevers *et al*, 2017). Furthermore, cells of the same cell type in different states may be detected in separate clusters. For the above reasons, it is best to use the term “cell identities” rather than “cell types”. Before clustering and annotating clusters, the user must decide which level of annotation detail, and thus which cluster resolution, is of interest.

**Figure 6:**
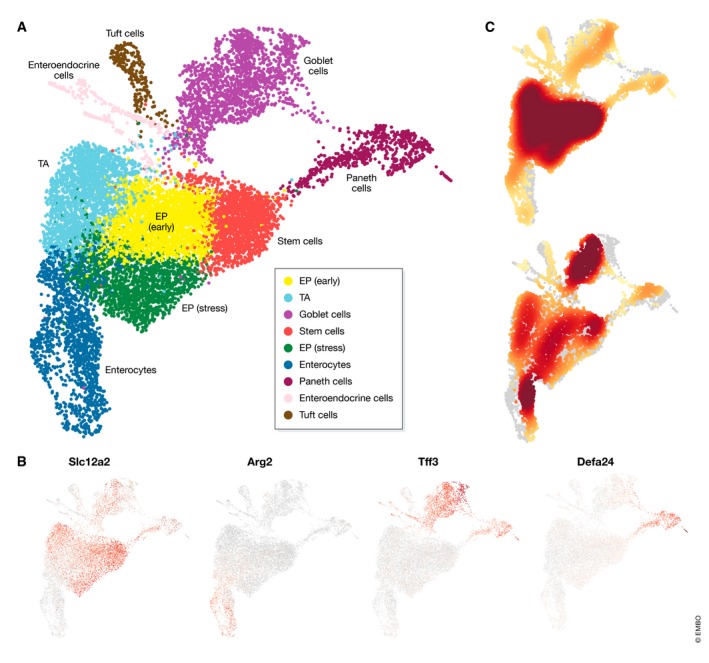
Cluster analysis results of mouse intestinal epithelium dataset from Haber *et al* (2017) (A) Annotated cell‐identity clusters found by Louvain clustering visualized in a UMAP representation. (B) Cell‐identity marker expression to identify stem cells (Slc12a2), enterocytes (Arg2), goblet cells (Tff3) and Paneth cells (Defa24). Corrected expression levels are visualized from low expression (grey) to high expression (red). Marker genes may be expressed also in other cell‐identity populations as shown for goblet and Paneth cells. (C) Cell‐identity composition heat maps of proximal (upper) and distal (lower) intestinal epithelium regions. High relative cell density is shown as dark red.

Identifying and annotating clusters relies on using external sources of information describing the expected expression profiles of individual cell identities. Thanks to recent and ongoing efforts such as the mouse brain atlas (Zeisel *et al*, 2018) or the Human Cell Atlas (Regev *et al*, 2017), reference databases are increasingly becoming available. These databases greatly facilitate cell identity annotation. In the absence of a relevant reference database, cell identities can be annotated by comparing data‐derived marker genes with marker genes from the literature (see case study on project github) or by directly visualizing the expression values of literature‐derived marker genes (Fig 6B). It should be noted that the latter method constrains the user to the classical understanding of cell types derived from bulk expression studies, rather than cell identities. Furthermore, it has been shown that commonly used cell surface markers are limited in their ability to define cell identities (Tabula Muris Consortium *et al*, 2018).

There are two ways to use reference database information to annotate clusters: using data‐derived marker genes or using full gene expression profiles. Marker gene sets can be found by applying differential expression (DE) testing between two groups: the cells in one cluster and all other cells in the dataset (see “Differential expression testing”). Typically, we focus on genes that are up‐regulated in the cluster of interest. As marker genes are expected to have strong differential expression effects, simple statistical tests such as the Wilcoxon rank‐sum test or the *t*‐test are often used to rank genes by their difference in expression between these two groups. The top‐ranked genes from the respective test statistic are regarded as marker genes. Clusters can be annotated by comparing marker genes from the dataset and marker genes from reference dataset via enrichment tests, the Jaccard index or other overlap statistics. Reference webtools such as www.mousebrain.org (Zeisel *et al*, 2018) or http://dropviz.org/ (Saunders *et al*, 2018) allow users to visualize the expression of dataset marker genes in the reference dataset to facilitate cell‐identity annotation.

Two aspects should be noted when detecting marker genes. Firstly, the *P*‐values obtained for marker genes are based on the assumption that the obtained cell clusters represent the biological ground truth. If one considers that there is uncertainty in the cluster assignment, the relationship between cluster assignment and marker gene detection must be accounted for in the statistical test. This relationship arises as both clusters and marker genes are typically determined based on the same gene expression data. The null hypothesis implicit in DE tests is that genes have the same distribution of expression values between the two groups. Yet, as the two groups are defined by the output of a clustering method in marker gene detection, there are differences in their gene expression profiles by design. We thus find significant marker genes even when clustering random data generated by splatter (Zappia *et al,* 2017) (see Appendix Supplementary Text S3). To obtain an appropriate measure of significance in clustered data, one can use a permutation test to account for the clustering step. This test is elaborated on in Appendix Supplementary Text S3. A recent differential expression tool also specifically addresses this issue (preprint: Zhang *et al*, 2018). With the current set‐up, the *P*‐values are often inflated, which can lead to an overestimation of the number of marker genes. However, the ranking of genes based on *P*‐values is unaffected. Assuming the clustering is biologically meaningful, the top‐ranked marker genes will still be the best marker gene candidates. In the first instance, we can loosely verify marker genes via visual inspection. We emphasize that inflated *P*‐values occur specifically when defining cell‐identity clusters via an unsupervised clustering approach. When instead determining cell‐identity clusters via the expression of individual genes, the *P*‐values can be interpreted as expected for all other genes. This univariate approach to cluster annotation, although common, is however not recommended outside specific cases (e.g. insulin in beta cells or haemoglobin in erythrocytes). Secondly, marker genes differentiate a cluster from others in the dataset and are thus dependent not only on the cell cluster, but also on the dataset composition. If the dataset composition does not accurately represent background gene expression, detected marker genes will be biased towards what is missing. This aspect must be considered especially when computing marker genes for datasets with low cellular diversity.

Recently, automated cluster annotation has become available. By directly comparing the gene expression profiles of annotated reference clusters to individual cells, tools such as scmap (Kiselev *et al*, 2018b) or Garnett (preprint: Pliner *et al*, 2019) can transfer annotations between the reference and the dataset. Thus, these methods can perform annotation and cluster assignment simultaneously, without the need for a data‐driven clustering step. As cell type and state compositions differ between experimental conditions (Segerstolpe *et al*, 2016; Tanay & Regev, 2017), clustering based on reference data should not replace the data‐driven approach.

The iteration of clustering, cluster annotation, re‐ or subclustering and re‐annotation can be time‐consuming. Automated cluster annotation methods offer a vast speedup of this process. However, there are benefits and limitations to automated and manual approaches that make it difficult to recommend one over the other. Increases in speed go together with compromises in flexibility. As mentioned above, reference atlases will not contain exactly the same cell identities as the dataset under investigation. Thus, one should not forgo marker gene calculation for manual annotation. Especially for large datasets that contain many clusters, the current best practice is a combination of both approaches. In the interest of speed, automated cell‐identity annotation can be used to coarsely label cells and identify where subclustering may be needed. Subsequently, marker genes should be calculated for the dataset clusters and compared to known marker gene sets from the reference dataset or literature. For smaller datasets and datasets that lack reference atlases, manual annotation will suffice.

**Pitfalls & recommendations:**

- Do not use marker gene *P*‐values to validate a cell‐identity cluster, especially when the detected marker genes do not help to annotate the community. *P*‐values may be inflated.
- Note that marker genes for the same cell‐identity cluster may differ between datasets purely due to dataset cell type and state compositions.
- If relevant reference atlases exist, we recommend using automated cluster annotation combined with data‐derived marker‐gene‐based manual annotation to annotate clusters.

#### Compositional analysis

At the cell level, we can analyse clustered data in terms of its compositional structure. Compositional data analysis revolves around the proportions of cells that fall into each cell‐identity cluster. These proportions can change in response to disease. For example, salmonella infection has been shown to increase the proportion of enterocytes in the mouse intestinal epithelium (Haber *et al*, 2017).

Investigating compositional changes in single‐cell data requires sufficient cell numbers to robustly assess cell‐identity cluster proportions, and sufficient sample numbers to evaluate expected background variation in cell‐identity cluster compositions. As appropriate datasets have only recently become available, dedicated tools are yet to be developed. In the mouse study mentioned above, cell‐identity counts were modelled using a Poisson process, including the condition as a covariate, and the total number of cells detected as an offset. Here, a statistical test can be performed over the regression coefficient to assess whether a particular cell identity has significantly changed in frequency. However, tests over other cell identities in the same dataset are not independent of each other. If the proportion of one cell‐identity cluster changes, the proportions of all others must have changed as well. Thus, one cannot assess whether the overall composition has significantly changed using this model. In the absence of dedicated tools, visual comparison of compositional data can be informative of changes in compositions between samples (Fig 6C). Future developments in this field will likely borrow from the mass cytometry (e.g. Tibshirani *et al*, 2002; Arvaniti & Claassen, 2017; Lun *et al*, 2017; Weber *et al*, 2018) or the microbiome literature (Gloor *et al*, 2017), where compositional data analysis has received more attention.

**Pitfalls & recommendations:**

- Consider that statistical tests over changes in the proportion of a cell‐identity cluster between samples are dependent on one another.

### Trajectory analysis

#### Trajectory inference

Cellular diversity cannot sufficiently be described by a discrete classification system such as clustering. The biological processes that drive the development of the observed heterogeneity are continuous processes (Tanay & Regev, 2017). Thus, in order to capture transitions between cell identities, branching differentiation processes, or gradual, unsynchronized changes in biological function, we require dynamic models of gene expression. This class of methods is known as trajectory inference (TI).

**Figure 7:**
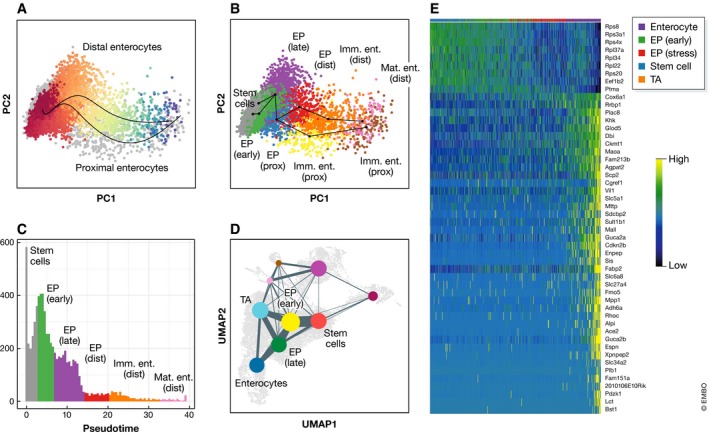
Trajectory analysis and graph abstraction of mouse intestinal epithelium data from Haber *et al* (2017) (A) Distal and proximal enterocyte differentiation trajectories inferred by Slingshot. The Distal lineage is shown coloured by pseudotime from red to blue. Other cells in the dataset are grey. (B) Slingshot trajectories over clusters in PCA space. Clusters are abbreviated as follows: EP—enterocyte progenitors; Imm. Ent.—immature enterocytes; Mat. Ent.—mature enterocytes; Prox.—proximal; Dist.—distal. (C) Density over pseudotime for the distal enterocyte trajectory from Fig 7A. Colours represent the dominant cluster labels in each pseudotime bin. (D) Abstracted graph representation of the dataset projected onto a UMAP representation. Clusters are shown as coloured nodes. Clusters that appear in other trajectories are labelled for comparison. “TA” denotes transit amplifying cells. (E) Gene expression dynamics over pseudotime in a general enterocyte trajectory using the “GAM” R library.

Trajectory inference methods interpret single‐cell data as a snapshot of a continuous process. This process is reconstructed by finding paths through cellular space that minimize transcriptional changes between neighbouring cells (Fig 7A and B). The ordering of cells along these paths is described by a *pseudotime* variable. While this variable is related to transcriptional distances from a root cell, it is often interpreted as a proxy for developmental time (Moignard *et al*, 2015; Haghverdi *et al*, 2016; Fischer *et al*, 2018; Griffiths *et al*, 2018).

Since Monocle (Trapnell *et al*, 2014) and Wanderlust (Bendall *et al*, 2014) established the TI field, the number of available methods has exploded. Currently available TI methods differ in the complexity of the paths that are modelled. Models range from simple linear or bifurcating trajectories, to complex graphs, trees, or multifurcating trajectories. In a recent comprehensive comparison of TI methods (Saelens *et al*, 2018), it was concluded that no individual method performs optimally for all types of trajectories. Instead, TI methods should be selected based on the complexity of the expected trajectory. The comparison revealed that Slingshot (Street *et al*, 2018) outperformed other methods for simple trajectories that range from linear to bi‐ and multifurcating models. If more complex trajectories are expected, PAGA (Wolf *et al*, 2019) was recommended by the authors. If the exact trajectory model is known, one can alternatively use more specialized methods to improve performance (Saelens *et al*, 2018). Generally, any inferred trajectory should be confirmed with an alternative method to avoid method bias.

In a typical workflow, TI methods are applied to reduced data or to corrected data when there is an inbuilt dimensionality reduction step. As multiple biological processes are typically occurring simultaneously within cells, it may be useful to regress out the biological effects of other processes to isolate the expected trajectory. For example, T cells may be undergoing cell cycle transitions during maturation (Buettner *et al*, 2015). Furthermore, as several top‐performing TI methods rely on clustered data, TI is typically performed after clustering. Clusters in inferred trajectories may represent stable or metastable states (see “Metastable states”; Fig 7B and C). Subsequently, RNA velocities can be overlayed onto the trajectory to add directionality (La Manno *et al*, 2018).

Inferred trajectories do not have to represent biological processes. In the first instance, these only denote transcriptional similarity. Few TI methods include an evaluation of uncertainty in their model (Griffiths *et al*, 2018). Thus, further information is needed to validate whether a biological process was indeed captured. This information can come in the form of perturbation experiments, inferred regulatory gene dynamics, and support from RNA velocity.

**Pitfalls & recommendations:**

- We recommend using the Saelens *et al* (2018) review as a guide.
- Inferred trajectories do not have to represent a biological process. Further sources of evidence should be collected to interpret a trajectory.

#### Gene expression dynamics

One approach to garner support that an inferred trajectory is not the result of fitting transcriptional noise is to analyse the trajectory on the gene level. Genes that vary smoothly across pseudotime characterize the trajectory and can be used to identify the underlying biological process. Furthermore, this group of trajectory‐associated genes is expected to contain genes that regulate the modelled process. Regulator genes help us understand how and why biological processes are triggered and represent potential drug targets (Gashaw *et al*, 2012).

While early approaches to find trajectory‐associated genes involved DE testing between cell clusters along a trajectory (Haghverdi *et al*, 2016; Alpert *et al*, 2018), we now detect genes that vary across a trajectory by regressing gene expression against pseudotime. In order to enforce smooth variation of expression along this covariate, pseudotime is smoothed by fitting a spline or via an additional local regression step (e.g. loess). Regression frameworks differ in their noise model assumptions and the class of function used to describe the expression as a function of pseudotime. Potential regulatory genes are obtained by performing model selection for the genes’ dependence on pseudotime. This DE test over pseudotime is confounded by the trajectory inference method in the same way that DE testing between clusters is confounded by the clustering method (see “Cluster annotation” section). Thus, *P*‐values obtained in this set‐up should not be regarded as an evaluation of significance.

Currently few dedicated gene temporal dynamics tools exist. BEAM is a tool integrated into the Monocle TI pipeline (Qiu *et al*, 2017a), which allows for detection of branch‐specific gene dynamics. Outside of this pipeline, users can opt for LineagePulse (https://github.com/YosefLab/LineagePulse), which considers dropout noise but is still in development, or write their own testing framework using the limma package (Ritchie *et al*, 2015) or standard R libraries. An example of this can be found in the online Slingshot tutorial (Street *et al*, 2018) and in Fig 7E.

Given the few available tools, a best practice for investigating gene temporal dynamics cannot yet be determined. Exploratory investigation of gene dynamics is surely possible using all above methods. In future, Gaussian processes may provide a natural model to investigate gene temporal dynamics. Furthermore, testing for regulatory modules rather than individual genes would likely improve the signal‐to‐noise ratio and facilitate the biological interpretation.

#### Metastable states

Cell‐level analysis of trajectories investigates cellular densities across pseudotime. Assuming that cells were sampled in an unbiased manner, dense regions along a trajectory indicate preferred transcriptomic states. When interpreting the trajectory as a temporal process, these dense regions may represent metastable states in, for example, development (Haghverdi *et al*, 2016). We can find these metastable states by plotting histograms of the pseudotime coordinate (Fig 7C).

### Cell‐level analysis unification

Clustering and trajectory inference represent two distinct views of single‐cell data. These two views can be reconciled in coarse‐grained graph representations. By representing single‐cell clusters as nodes, and trajectories between the clusters as edges, one can represent both the static and dynamic nature of the data. This unification was proposed by the partition‐based graph abstraction tool (PAGA; Fig 7D; Wolf *et al*, 2019). Using a statistical model for cell cluster interactions, PAGA places an edge between cluster nodes whose cells are more similar than expected. PAGA has been favourably compared to other TI methods in a recent review (Saelens *et al*, 2018). It was the only reviewed method able to cope with disconnected topologies and complex graphs containing cycles. This feature makes PAGA a helpful tool to visualize the topology of the entire dataset also for exploratory analysis.

### Gene‐level analysis

While we have so far focused on gene‐level analysis methods that characterize cellular structures, gene‐level analysis of single‐cell data has a broader scope. Differential expression testing, gene set analyses and gene regulatory network inference directly investigate molecular signals in the data. Rather than describing the cellular heterogeneity, these approaches use this heterogeneity as context in which gene expression is to be understood.

#### Differential expression testing

A common question asked of expression data is whether any genes are differentially expressed between two experimental conditions. DE testing is a well‐documented problem that originates from bulk gene expression analysis (Scholtens & von Heydebreck, 2005). An advantage over bulk differential testing is that we can account for cellular heterogeneity in the single‐cell setting by performing tests within cell‐identity clusters. This set‐up tells us how individual cell identities react transcriptionally under particular experimental conditions (Kang *et al*, 2018).

Although designed to answer the same question, bulk and single‐cell DE tools differ methodologically. While bulk methods were developed to accurately estimate gene variance from few samples, single‐cell data do not present this problem. On the other hand, single‐cell data contain unique technical noise artefacts such as dropout, and high cell‐to‐cell variability (Hicks *et al*, 2017; Vallejos *et al*, 2017). These artefacts are taken into account in methods designed specifically for single‐cell data (Kharchenko *et al*, 2014; Finak *et al*, 2015). Yet, a recent, large‐scale comparison study of DE analysis has suggested that bulk DE testing packages perform comparably to the best‐performing single‐cell tools (Soneson & Robinson, 2018). Furthermore, when bulk tools are adapted to model single‐cell data via introducing gene weights into the tests, these tools have been suggested to outperform their single‐cell counterparts (Van den Berge *et al*, 2018). According to this comparison, the top‐performing DE analysis tools are DESeq2 (Love *et al*, 2014) and EdgeR (Robinson *et al*, 2010) in combination with weights estimated by ZINB‐wave (Risso *et al*, 2018). Independent comparison studies that include weighted bulk DE testing methods are required to confirm these results.

The improved performance of weighted bulk DE testing comes at the cost of computational efficiency. Given the trend of increasing cell numbers in single‐cell experiments, algorithm runtime is becoming an increasingly important consideration in method choice. Thus, the single‐cell tool MAST (Finak *et al*, 2015) represents a potent alternative to weighted bulk DE tools. MAST uses a hurdle model to account for dropout while modelling changes in gene expression dependent upon condition and technical covariates. It was the best‐performing single‐cell DE testing method in the aforementioned study (Soneson & Robinson, 2018), and outperformed bulk and single‐cell methods in a small‐scale comparison on a single dataset (Vieth *et al*, 2017). While MAST has a 10‐fold to 100‐fold faster runtime than weighted bulk methods (Van den Berge *et al*, 2018), a further 10‐fold speedup can be achieved using limma–voom (Law *et al*, 2014). Although limma is a bulk DE testing method, limma–voom was shown to achieve comparable performance to MAST.

As uncorrected, measured data should be used for DE testing, accounting for confounding factors is crucial to robust estimation of differentially expressed genes. While DE testing tools typically allow the user the flexibility to incorporate confounders, users must be vigilant which variables are added to the model. For example, in most single‐cell experimental set‐ups the sample and condition covariates are confounded, since it is rarely possible to obtain a single sample under multiple conditions. If we incorporate both the sample and condition covariates into the model, the variability associated with these covariates can no longer unambiguously be assigned. Thus, when testing over condition, we cannot include the sample covariate in the model in the given form. When correcting for multiple categorical batch covariates, it becomes increasingly difficult to visually spot confounding groups of covariates. In this situation, it is helpful to test whether the model design matrix is full rank. Even when design matrices are not full rank, DE testing tools will often adapt the matrix and run without outputting a warning. This will not deliver the intended results.

In the scenario we describe here, the condition covariate is determined in the experimental set‐up. Thus, a DE test over this covariate (within the same cluster) is independent of the clustering procedure. This set‐up distinguishes DE testing over conditions and DE testing over clusters. Obtained *P*‐values for DE tests over conditions represent the expected measures of significance and must be corrected for multiple testing. To reduce the multiple testing burden, transcripts that may not be of interest can be excluded from the dataset. While pseudogenes or non‐coding RNAs can be informative (An *et al*, 2017), they are often ignored in the analysis.

**Pitfalls & recommendations:**

- DE testing should not be performed on corrected data (denoised, batch corrected, etc.), but instead on measured data with technical covariates included in the model.
- Users should not rely on DE testing tools to correct models with confounded covariates. Model specification should be performed carefully ensuring a full‐rank design matrix.
- We recommend using MAST or limma for DE testing.

#### Gene set analysis

Gene‐level analysis methods often produce long lists of candidate genes that are difficult to interpret. For example, thousands of genes may be differentially expressed between treated and control cells. We can facilitate the interpretation of these results by grouping the genes into sets based on shared characteristics and testing whether these characteristics are overrepresented in the candidate gene list.

Gene set information can be found in curated label databases for various applications. To interpret DE results, we typically group genes based on involvement in common biological processes. Biological process labels are stored in databases such as MSigDB (Liberzon *et al*, 2011), the Gene Ontology (Ashburner *et al*, 2000; The Gene Ontology Consortium, 2017), or the pathway databases KEGG (Kanehisa *et al*, 2017) and Reactome (Fabregat *et al*, 2018). Enrichment of annotations on the gene list can be tested using a vast array of tools, which are reviewed and compared in Huang *et al* (2009) and Tarca *et al* (2013).

A recent development in the single‐cell analysis field is the use of paired gene labels to perform ligand–receptor analysis. Here, interaction between cell clusters is inferred from the expression of receptors and their cognate ligands. Ligand–receptor pair labels can be obtained from the recent CellPhoneDB (Vento‐Tormo *et al*, 2018) and used to interpret the highly expressed genes across clusters using statistical models (Zepp *et al*, 2017; Zhou *et al*, 2017; Cohen *et al*, 2018; Vento‐Tormo *et al*, 2018).

#### Gene regulatory networks

Genes do not function independently. Instead, the expression level of a gene is determined by a complex interplay of regulatory interactions with other genes and small molecules. Uncovering these regulatory interactions is the goal of gene regulatory network (GRN) inference methods.

Gene regulatory network inference is performed based on measurements of gene co‐expression such as correlation, mutual information, or via regression models (Chen & Mar, 2018). If two genes show a co‐expression signal even when all other genes are taken into account as potential confounders, these genes are said to have a causal regulatory relationship. Inferring gene regulatory relationships is related to the detection of trajectory‐associated regulatory genes. Indeed, several single‐cell GRN inference methods use trajectories with mechanistic differential equation models (Ocone *et al*, 2015; Matsumoto *et al*, 2017).

While there exist GRN inference methods that were specifically developed for scRNA‐seq data (SCONE: Matsumoto *et al*, 2017; PIDC: Chan *et al*, 2017; SCENIC: Aibar *et al*, 2017), a recent comparison has shown both bulk and single‐cell methods to perform poorly on these data (Chen & Mar, 2018). GRN inference methods may still offer valuable insights to identify causal regulators of biological processes, yet we recommend that these methods be used with care.

**Pitfalls & recommendations:**

- Users should be wary of uncertainty in the inferred regulatory relationships. Modules of genes that are enriched for regulatory relationships will be more reliable than individual edges.

## Analysis platforms

Single‐cell analysis workflows are collations of independently developed tools. To facilitate the movement of data between these tools, single‐cell platforms have been developed around consistent data formats. These platforms provide a basis for the construction of analysis pipelines. Currently available platforms exist on the command line in R (McCarthy *et al*, 2017; Butler *et al*, 2018) or Python (Wolf *et al*, 2018), and as local applications (Patel, 2018; preprint: Scholz *et al*, 2018) or Web servers (Gardeux *et al*, 2017; Zhu *et al*, 2017) with graphical user interfaces (GUIs). An overview of platforms is available in Zhu *et al* (2017) and Zappia *et al* (2018).

Among command line platforms, Scater (McCarthy *et al*, 2017) and Seurat (Butler *et al*, 2018) easily interface with the large variety of analysis tools available via the R Bioconductor project (Huber *et al*, 2015). Scater has a particular strength in QC and pre‐processing, while Seurat is arguably the most popular and comprehensive platform, which includes a large array of tools and tutorials. A recent addition to this group is scanpy (Wolf *et al*, 2018), a growing Python‐based platform, which exhibits improved scaling to larger numbers of cells. It leverages the increasing number of tools written in Python, which is particularly popular for machine learning applications.

Graphical user interface platforms enable non‐expert users to build single‐cell analysis workflows. Users are often guided through prescribed workflows that facilitate the analysis, but also limit user flexibility. These platforms are especially useful for exploratory analysis. Platforms such as Granatum (Zhu *et al*, 2017) and ASAP (Gardeux *et al*, 2017) differ in the tools they integrate, with Granatum including the larger variety of methods. As Web servers, these two platforms are readily available, yet computational infrastructure will limit their ability to scale to large datasets. For example, ASAP was tested on a dataset of only 92 cells. Alternatives to the Web‐based GUI platforms are packages such as FASTGenomics (preprint: Scholz *et al*, 2018), iSEE (Rue‐Albrecht *et al*, 2018), IS‐CellR (Patel, 2018), and Granatum run on a local server. These are platforms and GUI wrappers that can scale with the locally available computational power. In future, the ongoing development of the Human Cell Atlas portals (https://www.humancellatlas.org/data-sharing) will lead to more powerful visual data exploration tools that scale to large cell numbers.

## Conclusions and outlook

We have reviewed the steps of a typical scRNA‐seq analysis workflow and implemented these in a case study tutorial (https://www.github.com/theislab/single-cell-tutorial). The tutorial was designed to follow current best practices as determined by available method comparisons. While aggregating individual best‐practice tools does not guarantee an optimal pipeline, we hope that our workflow represents a current snapshot of the state of the art in the single‐cell analysis field. It thus provides a suitable entry point into this field for newcomers and contributes to the efforts of the Human Cell Atlas to establish best practices in scRNA‐seq analysis (preprint: Regev *et al*, 2018). It should be noted that available method comparisons necessarily lag behind the latest method developments. Thus, we have mentioned new developments that have not yet been independently evaluated where possible. With the future development of new and better tools, and further comparative studies, the individual tool recommendations presented here will require updates, yet the general considerations regarding the stages of data processing should remain the same.

Two avenues of development that are of particular interest due to their potential for disruption to analysis pipelines are deep learning workflows and single‐cell omic integration. Due to its flexibility to scale to large data, deep learning has revolutionized fields from computer vision to natural language processing, and is starting to have a strong impact in genomics (Webb, 2018). First applications to scRNA‐seq are starting to emerge from dimensionality reduction to denoising (e.g. scVis: Ding *et al*, 2018; scGen: preprint: Lotfollahi *et al*, 2018; DCA: Eraslan *et al*, 2019). Recently, deep learning has been used to produce an embedded workflow that can fit the data, denoise it and perform downstream analysis such as clustering and differential expression within the framework of the model (scVI: Lopez *et al*, 2018). In this set‐up, it is possible to include noise and batch effect estimates into downstream statistical tests while preserving accurate estimates of variation in the data. Integrated modelling approaches such as this have the potential to replace current pipelines, which are often an agglomeration of individual tools.

As single‐cell omic technologies improve, the need for integrated omic analysis pipelines will grow (Tanay & Regev, 2017). Future single‐cell platforms will have to be able to deal with different data sources such as DNA methylation (Smallwood *et al*, 2014), chromatin accessibility (Buenrostro *et al*, 2015), or protein abundance (Stoeckius *et al*, 2017), and include tools that integrate these modalities. For this set‐up, it will no longer be possible to use only a single read or count matrix, which we use as the starting point of our tutorial. However, platforms are already adapting to multi‐modal data structures for the integration of RNA velocity, which is calculated from unspliced and spliced read data (La Manno *et al*, 2018). Single‐cell multi‐omic integration can occur via consensus clustering approaches, multi‐omic factor analysis (Argelaguet *et al*, 2018), or multi‐omic gene regulatory network inference (Colomé‐Tatché & Theis, 2018). Analysis workflows with these capabilities will be the next stage of development. We envisage that such multi‐omic analysis workflows will build upon the foundation we have laid for scRNA‐seq.

## Author contributions

MDL reviewed the literature and wrote the paper, and FJT supervised the work and critically reviewed the manuscript. Both authors read and approved the final paper.

## Conflict of interest

The authors declare that they have no conflict of interest.

## Supporting information



Appendix

Expanded View Figures PDF

Dataset EV1
