# Supplementary material for: Current best practices in single‐cell RNA‐seq analysis: a tutorial
Source: Mol Syst Biol. 2019 Jun 19;15(6):e8746. doi: 10.15252/msb.20188746 (PMC6582955; doi:10.15252/msb.20188746)
Supplement: Supplementary file 1 — Appendix [file MSB-15-e8746-s001.pdf]

Current best practices in single-cell RNA-seq analysis: a tutorial

Malte D. Luecken[1], Fabian J. Theis[1,2]

[1] Institute of Computational Biology, Helmholtz Zentrum München, German Research Center for Environmental Health, Neuherberg 85764, Germany
[2] Department of Mathematics, Technische Universität München, Garching bei München 85748, Germany

Appendix

Appendix **1**
Appendix Supplementary Text 1: Glossary 2
Appendix Supplementary Text 2: Experimental QC metrics 4
Appendix Supplementary Text 3: Permutation test for marker gene P-values 5
Appendix Figure 1: Analysis of marker gene detection in clustered random data 6

Appendix Supplementary Text 1: Glossary

Ambient gene expression:

Gene expression measurements from mRNA that does not originate from the captured cell, but is attributed to cells that were damaged in the experimental processing of the sample. The mRNA from these damaged cells leaks into the single-cell suspension and is captured along with viable cells in library construction.

Batch correction:

Methods that correct for differences in gene expression arising from unwanted, technical perturbations related to differing environmental conditions between two or more measurement batches with similar cell-type and state compositions. Batch correction typically involves linear models.

Data integration:

Methods that correct for batch effects between datasets that differ in their cell-type and state compositions. Data integration methods use non-linear approaches.

Data peeking:

Informing data filtering or collection decisions based on the outcome of a statistical test with the aim of generating a statistically significant result. Data peeking boosts p-values artificially.

Denoising:

See *expression recovery*.

Expression recovery:

Methods that aim to remove noise from the data and infer gene expression values for technical dropouts.

Imputation:

See *expression recovery*.

Library construction:

Experimental procedure of generating barcoded cDNAs from cellular RNA in a single-cell suspension. Barcoded cDNAs are sequenced to obtain read data.

Marker genes:

Genes that characterize a single-cell identity cluster. Marker genes are typically overexpressed in the cluster cells compared to other cells in the dataset.

Pseudotime:

A quantity used to order cells along an inferred trajectory given a pre-defined starting point. Assuming the trajectory represents a biological process, pseudotime is interpreted as “a quantitative measure of progress through a biological process” (Trapnell *et al*, 2014).

Summarization:

Dimensionality reduction methods that aim to describe a dataset in as few dimensions as possible without prescribing a particular number as in visualization. Summarization methods are used to reduce the data to its essential components thereby removing technical noise and biological stochasticity.

Technical dropouts:

Zero count measurements in gene expression data although the gene is actually being expressed in the relevant cell. Technical dropouts occur due to sampling effects resulting from not every cellular RNA molecule being captured, reverse transcribed, and sequenced.

Visualization:

Dimensionality reduction methods that aim to optimally describe a dataset in two or three components, which can be plotted to produce a visual representation of the data.

Appendix Supplementary Text 2: Experimental QC metrics

The quality of a single-cell dataset determines the amount of QC that is necessary to be able to perform downstream analysis. Low quality datasets can make it difficult to identify any cellular population. We can obtain an indication of the quality of the dataset from experimental QC metrics. These metrics are calculated during the processing of read data to generate count matrices. While there can be many indications that a dataset is of low quality, we focus on only a few central quantities that are typically calculated for every dataset. We emphasize that the QC metric targets we set here cannot be regarded as hard thresholds as they will differ between experimental techniques and biological tissue. For example, blood is easier to process than brain tissue and will thus result in higher experimental QC metrics.

The data that are output directly from the experimental pipeline are read sequences. Each read consists of base calls ('A', 'T', 'C', or 'G') with an assigned quality score that reflects the uncertainty in the call. Read pre-processing involves trimming of read sequences to filter out any uncertain calls, leaving high quality reads. A popular quality metric for sequencing is the percentage of base calls that have a quality score of over 30 (Q30 score). This metric is plotted over position in the read to discern barcode reads (cellular barcode + UMI) and biological reads (the mRNA sequence). Datasets that have poor Q30 scores in the barcode read will not be able to be assigned to a cell and are thus filtered out, while poor biological read quality will result in reads that cannot be aligned to the reference genome. Typically one expects Q30 scores above 60-70% throughout the read with particularly high Q30 scores in the barcode read. Read alignment will become difficult when Q30 scores fall too far below this threshold.

A further read-based experimental quality metric is generated during alignment. As we are mostly interested in gene expression rather than reads that cover intergenic regions, a high proportion of reads that are mapped to exonic regions is a further indicator of a successful experiment. While this proportion is also dependent on the biological system, proportions over 40% are desirable as non-exonic reads often represent wasted sequencing effort.

A direct quantification of wasted sequencing effort across the whole read processing pipeline represents a further experimental QC metric. During read processing, reads are assigned to barcodes, and barcodes that are assigned sufficient reads are thought to contain the transcriptome of at least one cell. Empty barcodes, and their associated reads, are filtered out before alignment. The proportion of reads that remain in the dataset after count matrices have been generated denotes the proportion of successful sequencing that was done. This metric can also be calculated at the cellular level. Depending on the experimental protocol, it may be known (or can be estimated) how many cells were input into the experiment. If approximately the same number of cells are captured in the count matrix, one can assume the experiment was performed to a high standard. Naturally, some loss of cells is expected, and cell losses will depend on the experimental protocol. However, one can calibrate the expected loss of cells against replicate experiments to assess the quality of data from a particular sample.

Appendix Supplementary Text 3: Permutation test for marker gene P-values

In the “Cluster annotation” section we argued that p-values are likely to be inflated when calculating marker genes for clusters of cells. This argument is based on the dependency between the test covariate (clusters), and the tested variables (gene expression data). Clusters are defined based on gene expression data, which results in cellular gene expression profiles differing between clusters by design. Here, we show this p-value inflation in simulated data and suggest an alternative marker gene detection test.

Using the splatter package (Zappia *et al*, 2017), we simulated random single-cell gene expression data for a single cluster and without differentially expressed genes (1,000 cells, 10,000 genes; code available on the project github). As we have determined the simulation parameters, we know that there is no substructure present in this data. Hence, any partition of this dataset would be performed based on fluctuation in gene expression due to noise and not an underlying signal. After basic pre-processing (filtering cells and genes, CPM normalization, log-transformation, and HVG selection for top 4000 genes), and best-practices louvain clustering at a resolution of 0.5, we obtained 2 clusters (Appendix Figure 3a). Finally, we performed marker gene detection for these clusters using a t-test. The analysis script for this simulation is available at https://github.com/theislab/single-cell-tutorial/.

Although our clusters are only representations of the noise in the data, the distribution of p-values over all genes is skewed towards low p-values (Appendix Figure 3b). As p-values are uniformly distributed under null model conditions, the skewed p-value distribution shows us that our simulated data do not come from the null model. Yet, we have simulated conditions that can be regarded as random for single-cell RNA-seq data. Thus, differential expression tests between cell clusters are biased towards low p-values even under random conditions. Indeed, we find 5 and 9 marker genes with FDR-adjusted p-values below the significance threshold of 0.05 in this random dataset. As argued in the main text, the cause of this p-value inflation is the clustering step, which should be taken into account in the test statistic.

A simple method to take into account clustering into the differential testing null model is via a permutation test. By permuting the expression values per gene in a real gene expression dataset, we can generate a random dataset while conserving the distribution of expression values for each gene. After clustering the permuted data, we can compute marker genes for each cluster. The p-values of these marker gene tests are p-values that arise from random data after clustering. We propose to use these random data p-values as a background distribution against which we can evaluate the real data p-values obtained from our clustered real data. To obtain a p-value for a marker gene conditional upon clustering we can calculate an empirical p-value defined by $p_{emp} = \frac{m+1}{M+1}$. Here, m is the number of random data p-values lower than our real data p-value, and M represents the total number of random p-values in the distribution.

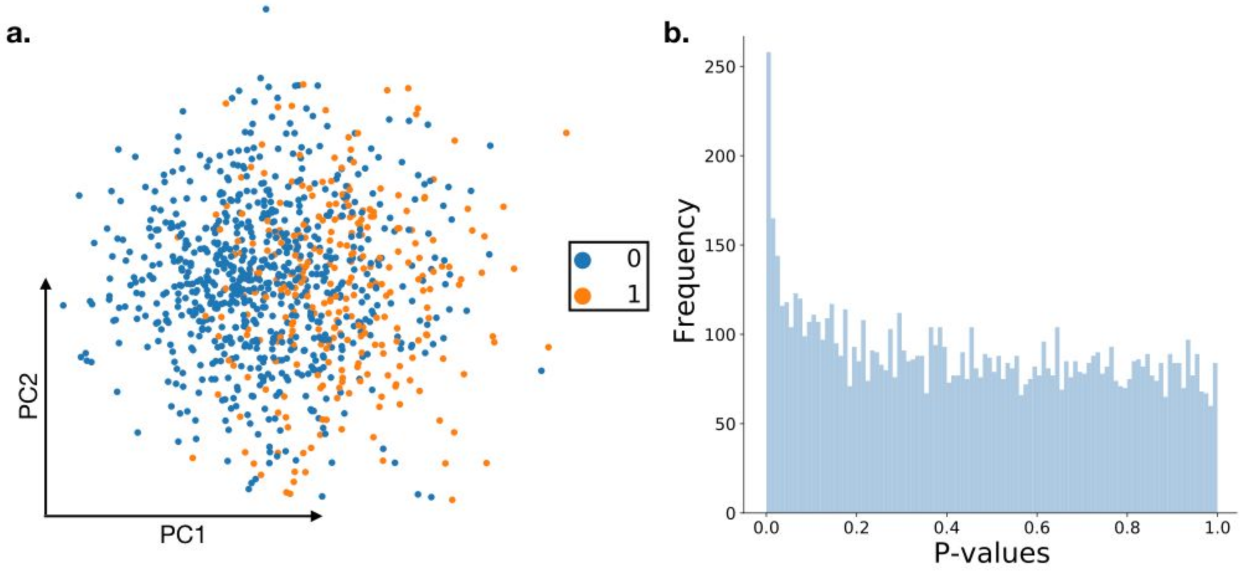

Appendix Figure 1: Analysis of marker gene detection in clustered random data. Random data were simulated using the splatter package for one cluster, with dropout, and without differentially expressed genes. After Louvain community detection we detected 2 clusters, which are visualized in PCA-space in **a.** Marker gene detection was performed in scanpy using the t-test. The distribution of the p-values of all genes is shown in **b.**

P-value distributions obtained from the random data will differ based on the size of the cluster and the rank of the p-value in the marker gene test. In order to account for these dependencies, we suggest that the background p-value distribution for each empirical p-value calculation should be taken from a subset of the total random p-value distribution. Specifically, we propose that permuted data clusters are binned by cluster size, and within these size bins, only p-values with the same rank in their cluster are used. For example, to calculate a empirical p-value for the top marker gene of a cluster of size 50, one should use the lowest p-value genes from all clusters of size 40-60 in the random data. To obtain robust assessments of empirical p-values with this filtering, it will be necessary to generate several thousand permuted datasets, which may make this test scale poorly to large numbers of cells. A recently proposed marker gene detection tool that addresses the same issue may provide a more computationally efficient solution (Zhang *et al*, 2018).
