# Supplementary material for: Current best practices in single‐cell RNA‐seq analysis: a tutorial
Source: Mol Syst Biol. 2019 Jun 19;15(6):e8746. doi: 10.15252/msb.20188746 (PMC6582955; doi:10.15252/msb.20188746)
Supplement: Supplementary file 2 — Expanded View Figures PDF [file MSB-15-e8746-s002.pdf]

Expanded View Figures

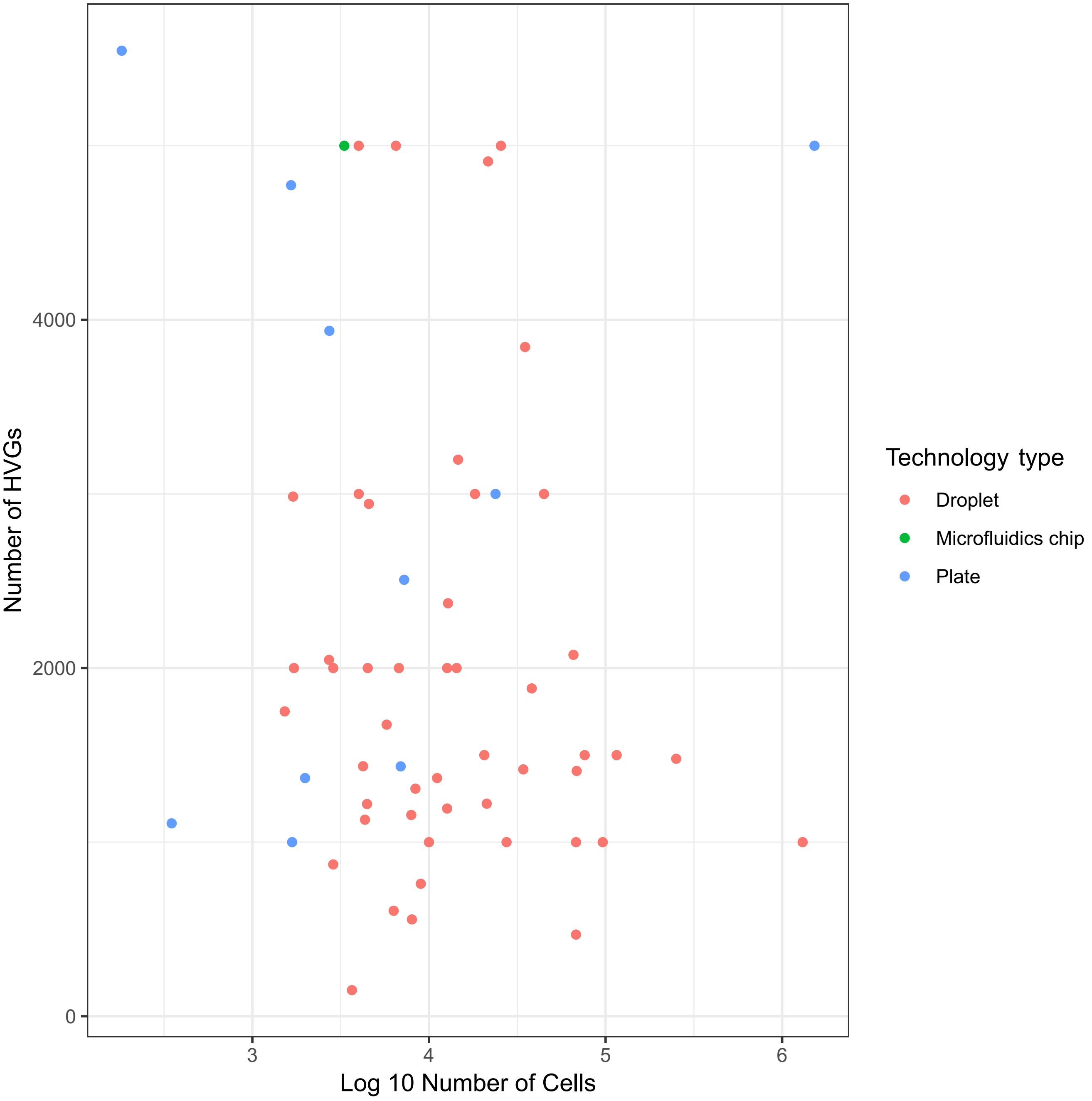

Figure EV1. The number of highly variable genes (HVGs) used for datasets of different sizes.
The data were obtained by a brief manual survey of recent scRNA-seq analysis papers. The plotted data, along with further information on scRNA-seq technology, publication year, reference and the number of reads per cell, are available in Dataset EV1.

 © 2019 The Authors

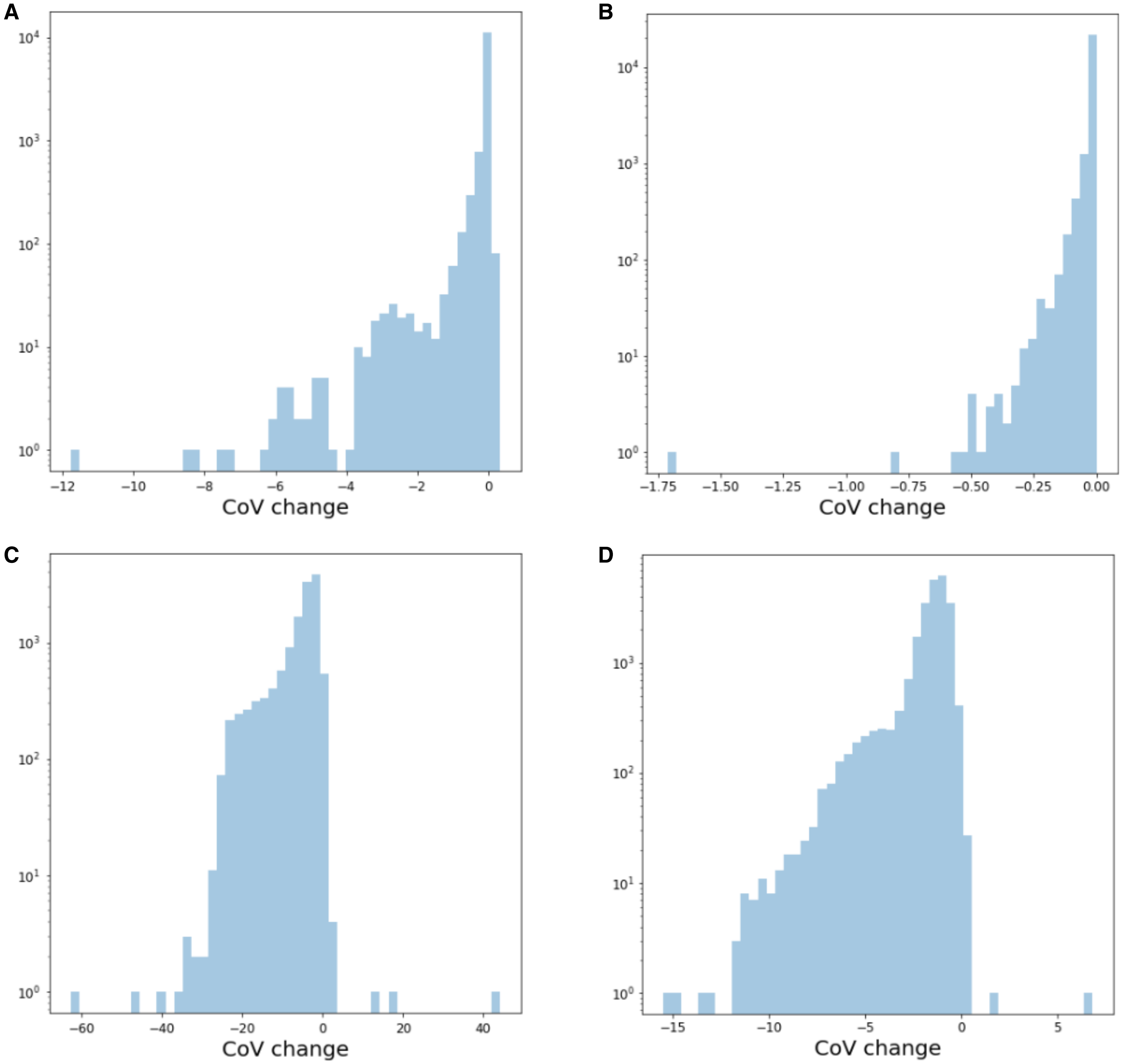

Figure EV2. Change in coefficient of variation (CoV) of gene expression data upon batch correction and denoising.
Negative values represent a reduction in CoV upon data correction. The top row shows CoV changes upon ComBat batch correction for (A) mouse intestinal epithelium (mIE) and (B) mouse embryonic stem cell (mESC) data. The lower row depicts CoV changes upon DCA denoising for (C) mIE and (D) mESC data. mIE data were obtained from Haber *et al* (2017) and mESC from Klein *et al* (2015).

© 2019 The Authors
